# Speech Perception in Older Hearing Impaired Listeners: Benefits of Perceptual Training

**DOI:** 10.1371/journal.pone.0113965

**Published:** 2015-03-02

**Authors:** David L. Woods, Zoe Doss, Timothy J. Herron, Tanya Arbogast, Masood Younus, Marc Ettlinger, E. William Yund

**Affiliations:** 1 Human Cognitive Neurophysiology Laboratory, VANCHCS, 150 Muir Rd., Martinez, CA, 95553, United States of America; 2 UC Davis Department of Neurology, 4860 Y St., Suite 3700, Sacramento, CA, 95817, United States of America; 3 Center for Neurosciences, UC Davis, 1544 Newton Ct., Davis, CA, 95616, United States of America; 4 UC Davis Center for Mind and Brain, 202 Cousteau Place, Suite 201, Davis, CA, 95616, United States of America; University Of Cambridge, UNITED KINGDOM

## Abstract

Hearing aids (HAs) only partially restore the ability of older hearing impaired (OHI) listeners to understand speech in noise, due in large part to persistent deficits in consonant identification. Here, we investigated whether adaptive perceptual training would improve consonant-identification in noise in sixteen aided OHI listeners who underwent 40 hours of computer-based training in their homes. Listeners identified 20 onset and 20 coda consonants in 9,600 consonant-vowel-consonant (CVC) syllables containing different vowels (/ɑ/, /i/, or /u/) and spoken by four different talkers. Consonants were presented at three consonant-specific signal-to-noise ratios (SNRs) spanning a 12 dB range. Noise levels were adjusted over training sessions based on d’ measures. Listeners were tested before and after training to measure (1) changes in consonant-identification thresholds using syllables spoken by familiar and unfamiliar talkers, and (2) sentence reception thresholds (SeRTs) using two different sentence tests. Consonant-identification thresholds improved gradually during training. Laboratory tests of d’ thresholds showed an average improvement of 9.1 dB, with 94% of listeners showing statistically significant training benefit. Training normalized consonant confusions and improved the thresholds of some consonants into the normal range. Benefits were equivalent for onset and coda consonants, syllables containing different vowels, and syllables presented at different SNRs. Greater training benefits were found for hard-to-identify consonants and for consonants spoken by familiar than unfamiliar talkers. SeRTs, tested with simple sentences, showed less elevation than consonant-identification thresholds prior to training and failed to show significant training benefit, although SeRT improvements did correlate with improvements in consonant thresholds. We argue that the lack of SeRT improvement reflects the dominant role of top-down semantic processing in processing simple sentences and that greater transfer of benefit would be evident in the comprehension of more unpredictable speech material.

## Introduction

Older hearing impaired (OHI) listeners exhibit deficits in consonant identification [1–5] that contribute to problems in understanding connected speech, including sentences. Hearing aids (HAs) improve consonant identification in OHI listeners [6], with the magnitude of HA benefit on consonant identification correlating with improvements in sentence reception thresholds (SeRTs) [7,8]. However, aided OHI listeners continue to show large deficits in consonant identification in comparison to older listeners with normal hearing (ONH), particularly for harder-to-identify consonants [7]. For example, when tested with large consonant sets aided OHI listeners with mild to moderately severe hearing losses identify only 60–75% of consonants even in quiet [9,10].

Traditionally, the failure of HAs to restore consonant identification to normal levels has been ascribed to “inaudibility”, i.e., the acoustic features that are critical for consonant identification remain below the listener’s audiometric threshold, even in aided listening conditions. However, recent studies have shown that observed HA benefits in consonant identification [11] and sentence processing [12] are smaller than those predicted by the increases in audibility provided by the HAs. This suggests that OHI listeners are unable to take full advantage of the speech cues made audible by their HAs.

Such findings have motivated a number of studies to investigate whether speech-discrimination training can enhance speech-cue utilization in aided OHI listeners. Different training programs have targeted training at sentence, word, and phoneme levels. However, the efficacy of speech-discrimination training remains to be established: a recent review [13] concluded that “published evidence for the efficacy of individual computer-based auditory training for adults with hearing loss is not robust and therefore cannot be reliably used to guide intervention at this time.” Among the challenges highlighted in the review were distinguishing between effects due to procedural and perceptual learning, the control of placebo effects, and the variability in outcomes measured among individual listeners.

### Sentenced-based training

Sentenced-based training targets the ultimate goal of audiological rehabilitation: improved understanding of conversational speech. Sweetow and Sabes [14] developed the sentence-based training program, Listening and Communication Enhancement (LACE), and evaluated its efficacy in 65 OHI listeners who trained for 20 days (30 min/day) on five different sentence-level tasks. Listeners’ performance significantly improved (by 3–4 dB speech-to-babble ratio) on the trained tasks, and listeners showed small but significant improvements in sentence reception thresholds (SeRTs) measured with the Quick Speech In Noise test (QSIN)[15], as well as a trend toward significant improvements of SeRTs measured with the Hearing In Noise Test (HINT) [16]. A recent study of 29 OHI listeners replicated these findings and reported somewhat larger improvements in new HA users [17]. However, a larger multi-center study [18] that compared LACE benefits in a group of 105 aided OHI listeners and 121 matched controls, all with mild-to-moderate hearing loss, showed improvements in LACE training tasks, but no training benefits on the Words in Noise (WIN) test or the Speech in Noise (SPIN) test [19]. Moreover, there were no significant correlations between the magnitudes of benefit seen during training and the magnitudes of benefit observed on the WIN and SPIN.

### Word, phrase, and phoneme-based training

Several studies have reported that word-based training improves speech recognition in cochlear implant (CI) patients. For example, Fu et al. [20] trained CI patients for one month on word discriminations that involved contrasting vowels (e.g., “seed” vs. “said”) or consonants (“cap” vs. “cat”). They found that improvements generalized to other vowel and consonant discriminations and, in three patients, generalized to improved sentence recognition. In a more recent study [21], CI patients were trained to recognize digits in speech babble. Training improved the identification of digits in both babble and speech-spectrum noise, and training benefit generalized to improvements in sentence recognition.

Humes and colleagues [22–26] performed a series of studies exploring word-based training in OHI listeners. They first found that training listeners to identify lists of 75 words produced large improvements in trained word thresholds, regardless of whether they were spoken by familiar or unfamiliar talkers. However, no improvements were found in the recognition of untrained words, or the recognition of trained words when presented in sentences [22]. Next, Burk and Humes [23] examined the effects of longer duration training (12 weeks) using 75 lexically hard words (infrequent words with high neighborhood densities) and 75 lexically easy words. Training benefits were set-specific, did not generalize to untrained words, and did not improve the recognition of trained words in sentence contexts. Humes and colleagues subsequently investigated word-training benefits in young normal hearing (YNH) and OHI listeners [25]. Since their previous results had suggested that training benefits were lexically based (i.e., trained-word identification improved, regardless of the talker), this study investigated the benefits of presenting 600 of the most common words in spoken American English and 94 frequently occurring American English phrases. Listeners received a total of 28–36 hours of training. Identification accuracy increased for trained words presented in isolation, and in sentences. However, in two more recent studies using a similar training protocol, training benefits did not generalize to improved word recognition in sentences [26,27].

### Multi-level training

Miller and Watson have developed the Speech Perception Assessment and Training System (SPATS) [28–30], which includes sentence and syllable-based training. Listeners select the words in the sentence from a closed set, while sentence SNRs are adjusted adaptively. In SPATS’ syllable-based training, listeners identify syllables that differ in onset and coda consonants, consonant clusters, and vowels. Listeners are asked to imitate the sound and then identify it, with visual feedback and optional sound replay. SNRs are adaptively based on the accuracy of closed-set responses. Identification accuracy improved by up to 16% (3–4 dB SNR) in early reports [28–30]. Large-scale clinical trials of SPATS are now underway [31].

### Training consonant identification

Consonants convey the majority of information in conversational speech, and consonant thresholds show large elevations in OHI listeners, even in aided listening conditions [7]. When high-frequency hearing is partially restored by HAs, consonant identification improves and consonant-cue weightings are partially normalized [7]. However, HAs produce smaller improvements in consonant identification than might be expected based on improvements of audibility [11]. One explanation is that compensatory neuroplastic changes have occurred in auditory cortex as hearing loss develops [32], including the pruning of connections transmitting high-frequency information [33–35]. Because high-frequency information is more severely compromised than low-frequency information in most OHI listeners they must rely disproportionately on imprecise low-frequency cues to discriminate consonants. For example, when discriminating fricatives, OHI listeners are unable to use high-frequency frication cues and instead use less-reliable duration [35] or overall consonant/vowel amplitude ratio cues [36].

The neural reorganization that occurs as hearing loss develops must be reversed in order for OHI listeners to more fully utilize the high-frequency information restored by HAs. However, the conditions needed to force the neuronal reorganization are rarely experienced. The Hebbian conditions needed to restore normal weightings of high-frequency cues require that high-frequency—but not low-frequency—cues, provide accurate information about consonant identity. Because HAs improve the audibility of both low- and high-frequency consonant cues, OHI listeners may continue to rely on low-frequency cues. While noisy listening conditions will mask low frequencies and force subjects to rely on high-frequency cues, OHI listeners tend to avoid such environments. Moreover, OHI listeners learning to use HA-restored cues would benefit from immediate feedback about the accuracy of consonant identification. Such feedback is absent in most conversational situations. As a result, the perceptual learning needed to restore the utilization of high-frequency consonant information rarely occurs.

However, such conditions can be imposed by training paradigms. For example, in a previous study we demonstrated that consonant-identification training in speech-spectrum noise resulted in significant improvements in consonant-identification performance in aided OHI listeners [37]. In that study, listeners underwent eight weeks (40 hours) of adaptive, PC-based consonant identification training in their homes, and showed an improvement of 9.8% in consonant-identification performance measured in the laboratory. Importantly, training with syllables spoken by one set of talkers improved consonant identification for syllables spoken by different talkers. Moreover, no significant performance decline was observed on retention testing two months after the end of training.

### Do training benefits reflect procedural learning, content learning, or perceptual learning?

Improved speech recognition due to training can be the result of procedural learning, content learning, or perceptual learning. Procedural learning reflects incidental improvements in performance that occur when listeners become familiar with a task. For example, SeRTs of inexperienced listeners, measured in quiet, may improve substantially as listeners learn to minimize self-generated noise (e.g., respiration) during testing [38,39]. Procedural learning is associated with improvements in the signal-detection metric of receiver sensitivity (d’). However, procedural learning occurs much more rapidly than perceptual learning, and, by definition, does not generalize beyond test conditions.

Content learning depends on the memory for specific items, such as the syllables, words, or sentences, presented during training. Content learning occurs rapidly and can be substantial. For example, SeRTs improve rapidly when the same sentence is presented repeatedly, and even a single presentation of sentences can alter SeRTs measured with the same sentences up to three months later [39]. Training improvements due to content learning do not index improvements in speech comprehension *per se* (i.e., improvements in d’), but rather reflect alterations in response criteria, β. Listeners are biased to report words used in training, at the expense of increased reports of trained words when other confusable words are presented (i.e., increased false alarms). For example, training a listener with a word set that includes the word “tot” will increase the likelihood of correct report when “pot” is presented, but will also an increase the likelihood of “pot” report when the listener is presented with syllables with which “tot” can be confused, such as “pop”, “tot”, “hop”, “top”, “hot”, “cot”, etc.

Insofar as some words occur much more frequently than others in English, it might be argued that biasing the report of frequent words would nevertheless be beneficial. However, imposing a “frequent-word” bias through training is problematic for several reasons. First, any preexisting listener bias, developed as a consequence of their everyday listening experience, would presumably be better suited to the speech that they experience on a daily basis than an artificial bias imposed by training. Second, with the exception of function words, it is difficult to categorize words on the basis of frequency of occurrence, as word-frequency counts vary considerably across different speech corpuses. For example, of the approximately 450 content words trained by Humes [25], more than 40% do not occur among the most common 600 words in film dialogs in the SUBTLEX-US corpus [40]. In addition, the frequency count of words declines gradually, making any decision about the number of words to include in training somewhat arbitrary. For example, in the SUBTLEX database words 600–700 occur 76% as often as words 500–600. As a result, the aggregate frequency of occurrence of the words that can be confused with a “frequent” word is often much higher than the frequency of occurrence of the frequent word itself. Thus, developing an increased bias for frequent word report might increase word misidentifications overall. Finally, frequency is generally inversely proportional to the semantic information conveyed by a lexical item [41]. Thus, while word identification rates may improve with a frequent-word bias, this will lead to higher errors rates for words that are more important to the conversation.

Changes in response bias may also occur in phoneme-based training paradigms due to statistical dependences of phoneme occurrence. For example, SPATS trains listeners to identify 45 onsets, 28 nuclei, and 36 coda [30]. However, the 388 tokens used in SPATS training represent less than 1% of all possible combinations of the phonemes included in the training set. As a result, there are inevitable statistical dependencies of phoneme occurrence that will bias report with training. For example, the onset /pl/ is trained in four syllables, including the token “pler”. Insofar as the phoneme string “ler” occurs more often in association with /p/ than with other plosives, subjects will tend to report “pler” more often in high noise situations, but misidentify “blur” and other similar syllables unless they are also trained.

In contrast to procedural learning and changes in report bias due to content learning, perceptual learning reflects true improvements in the fundamental ability of listeners to discriminate speech sounds. Perceptual learning is reflected by increases in d’, with or without alterations in report bias, β. Perceptual learning generally requires prolonged, adaptive training and has been associated with neuroplastic alterations in auditory cortex [42]. Depending on the range of materials used in training, perceptual learning may generalize to test conditions that differ substantially from those used in training [43–46].

The current experiment investigated perceptual learning of consonant identification in noise. The training paradigm optimized the conditions necessary for perceptual learning by (1) presenting multiple exemplars of consonants to be learned; (2) using conditions difficult enough to produce significant error rates; (3) increasing discrimination difficulty adaptively based on listener performance; (4) providing immediate feedback after each response; and (5) training for an extended (40-day) period [47]. A very large token set of consonant-vowel-consonant (CVC) syllables was used, based on evidence that improvements in phoneme discrimination generalize to a greater extent when training occurs in multiple phonemic contexts [48–51]. In order to rule out possible contributions from content learning, the syllables used in training were constructed from the exhaustive combination of all consonants and vowels included in the training set. As a result, the occurrence of each consonant was statistically independent from the occurrence of all other phonemes. This assured that any improvement in consonant-identification would reflect perceptual learning, instead of alterations in response bias due to content learning.

We also evaluated the extent to which consonant-identification training would improve SeRTs measured with the HINT and QSIN [15]. The SeRTs of OHI listeners are accurately predicted by the identification thresholds of easily identified consonants [7], and HA benefit on SeRTs is accurately predicted by the magnitude of HA benefit on easily identified consonant thresholds [7]. However, HA benefit on SeRTs is only a small fraction (about 13%) of HA benefit on of easy consonant thresholds. In the current study, we anticipated that training would improve consonant identification thresholds, and that training benefits on SeRTs would similarly depend on the magnitude of training benefits on easily identified consonants.

## Methods

### Overview

We first identified a number of areas in our previous training paradigm [37] that could be further improved. (1) In the previous study, SNRs were adjusted on a trial-by-trial basis and consonants were presented randomly. However, subsequent work demonstrated that the SNR thresholds needed to identify different consonants vary by more than 40 dB [52,53]. Thus, trial-to-trial performance adjustments based on accuracy would largely reflect the consonant that had been randomly selected, rather than any learning-related changes in consonant-identification ability. (2) While our previous training corpus included 18 consonants, only nine unvoiced consonants were trained at syllable onsets and nine voiced consonants were presented in the coda (syllable-ending) position. This limited stimulus set did not permit a complete analysis of consonant confusions; e.g., no information was available about voiced consonant confusions in the onset position or unvoiced consonant confusions in the coda position. (3) Only a single adjustable SNR was used in training, whereas multiple SNRs would be expected to enhance training generalization [54]. SNR variability would also reduce the possibility that listeners could use the intrinsic difficulty of consonant identification to improve guessing strategies. (4) Performance improvements were analyzed using percent correct-measures. Our subsequent studies have shown that listeners have different response criteria for different consonants [53], so that the signal detection parameter, d’, should be used when assessing changes in identification performance for individual consonants.

Therefore, in the current experiment, we introduced the following improvements: (1) Rather than adjusting SNRs on a trial-by-trial basis, gradual, sub-dB adjustments were made in the SNR of each consonant based on individual consonant-identification thresholds averaged over the previous six hours of training. Thus, SNR adjustments reflected the measured learning-related changes in the thresholds of each consonant. (2) The training corpus included 20 onset and 20 coda consonants; i.e., almost all commonly occurring American English consonants in each syllable position. (3) Listeners trained with consonant-vowel-consonant (CVC) syllables rather than CVs and VCs. This permitted onset and coda consonants to be trained simultaneously. (4) A very large set of tokens was created for training by exhaustively combining the 20 onset and 20 coda consonants with three different vowels to create 1,200 (20 x 20 x 3) unique syllables. Each syllable was then recorded twice by each of the four different talkers (two male, two female) to create a training corpus of 9,600 tokens. This resulted in substantial articulatory variability of the tokens used in in training. (5) Consonants were presented at three different SNRs spanning a 12 dB range. (6) The SNRs spanned a consonant-specific baseline value that had been adjusted to approximately equate consonant-identification difficulty for different consonants, so that easily identified consonants were presented at lower SNRs than more difficult consonants. (7) Performance was measured with d’ rather than percent correct measures.

### Ethics Statement

All listeners gave informed, written consent following procedures approved by the VANCHCS Institutional Review Board and were paid for their participation.

### Listeners

19 older male Veteran patients (mean age 70 years, range 61 to 81 years) with mild-to-moderate hearing loss were recruited. Listeners had been prescribed advanced digital HAs and had been using their current HAs for an average of 1.7 years (none less than 8 months). Because listeners had been recruited from an older Veteran population, all listeners were male. Of the 19 listeners recruited, two discontinued training. A third listener took a 4-week vacation during the middle of the 8-week training period and was therefore excluded from further analysis. The remaining 16 listeners completed 40 training sessions in 8 to 13 weeks. The trained group included 11 listeners who had been previously tested in unaided and aided conditions to evaluate hearing aid benefit [7].

**Fig 1:**
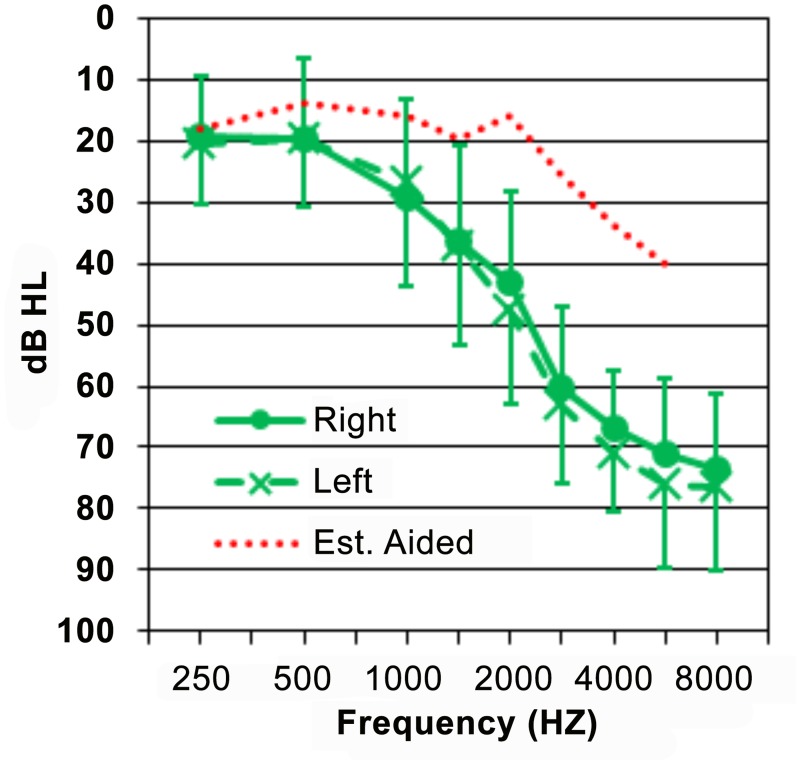
Average audiogram for the listeners who completed training and their average estimated aided thresholds based on HA prescriptive targets. Error bars show standard deviations.

OHI listeners were selected from patients fitted with HAs by the audiology service during the previous 6–18 month period, who had mild-to-moderately severe sloping hearing losses that were bilaterally symmetrical (within 15 dB, see Fig. 1). As a result, the OHI listeners had normal hearing to mild losses at 500 Hz (maximum 40 dB HL), gradually increasing to more severe losses at higher frequencies. This audiometric configuration is typical of older Veteran listeners with hearing loss and is similar to that of the populations used in previous perceptual training studies [25,28,37]. When appropriate, we compared their results to those of 16 older-normal hearing (ONH) listeners who had been previously studied with the same syllable and sentence materials [55].

All listeners were in good mental and physical health, had normal daily functioning, and spoke English as their native language. Listeners were recruited after an examination of their audiological records and medical history. Exclusion criteria included a history of dementia or mild cognitive impairment, chronic alcoholism or drug abuse, on-going treatment with ototoxic drugs or psychopharmaceutical agents, a history of neurological disorders or severe head trauma, or chronic recurrent disease.

### Pre- and post-training assessments

Prior to training, consonant identification in noise performance was measured using the California Syllable Test (CaST) [52,53]. Procedures used for CaST, HINT and QSIN testing have been described in detail in a previous study of unaided and aided OHI listeners [7]. All pre- and post-training testing was performed in aided conditions.

### Consonant Groups

As in previous studies of normal hearing listeners [52,53], consonants were divided into three groups based on their identifiability: Group A consonants (/t/, /s/, /ʃ/, /ʧ/, /z/, /ʤ/, and /r/) are identified by normal hearing listeners at SNRs similar to those of SeRTs (e.g. -2 dB), Group B consonants (/k/, /f/, /d/, /g/, /m/, /n/, and /l/) are identified at SNRs 10–12 dB above SeRTs, and Group C consonants (/p/, /ɵ/, /b/, /v/, /h/, /ð/, and /ŋ/) are identified at SNRs more than 20 dB above SeRTs. Consonants from Group A and Group B each constitute about 40% of consonant used in conversational speech, and consonants from Group C about 20%.

### In home training

Listeners identified 360 syllables during each daily at-home training session. To assure that listeners understood the training procedure, all listeners underwent at least one in-laboratory training session before beginning training at home. Training at home was performed with 19” Dell Studio One (all-in-one) computers with built-in speakers that were positioned in a quiet listening location in the home by a research associate. The speakers (located on each side of the monitor) were calibrated using a Bruel and Kjaer 2260 sound meter with Head and Torso System (HATS). Sound intensities were adjusted to be identical to those used during in-lab CaST testing. Spectral analysis showed a small reduction in low frequencies in comparison with the sound-delivery system used for in-laboratory testing, and a slightly flatter frequency response in the 2–4 kHz range. However, differences at any frequency were less than 3 dB.

Listeners trained for 1-hr/day, 5 days per week over several months. They were instructed to wear their HAs in fixed settings during training and testing sessions and sit approximately 0.8m directly in front of the monitor while training. During training, CVC intensities were roved from 70–75 dB SPL. Each training session consisted of two 1-hr segments (each containing 360 trials) performed on separate days. Thus, each training session involved the presentation of 720 syllables, which were balanced for talker (180 of each of four talkers), vowel (240 of each of three vowels), and SNR (240 at each of three baseline-relative SNRs).

Training included 4,800 tokens from the 9,600 CaST token set used for in-laboratory testing, and 4,800 CVC tokens, spoken by two different talkers, that were used only during training. Thus, two of the voices used in CaST testing became familiar over the course of training, whereas the other two voices used in the CaST remained unfamiliar. Training materials and software can be obtained at www.ebire.org/hcnlab/tools/hearing/CINT.

Initial baseline (B) levels were set based on the results of in-laboratory testing at study entry, and were adjusted over training days based on the listener’s performance. Specifically, the baseline SNR of each onset and coda consonant was increased or decreased after each training session to maintain a d’ of 2.20, based on a moving average of performance over the current and two previous training sessions. Thus, if performance improved, SNRs decreased, whereas if performance deteriorated, SNRs increased. However, since SNRs were truncated at a maximum baseline SNR of 40 dB for some consonants with high pre-training thresholds (e.g., many Group C consonants), performance on those consonants could improve substantially before the SNRs used in training began to change.

**Fig 2:**
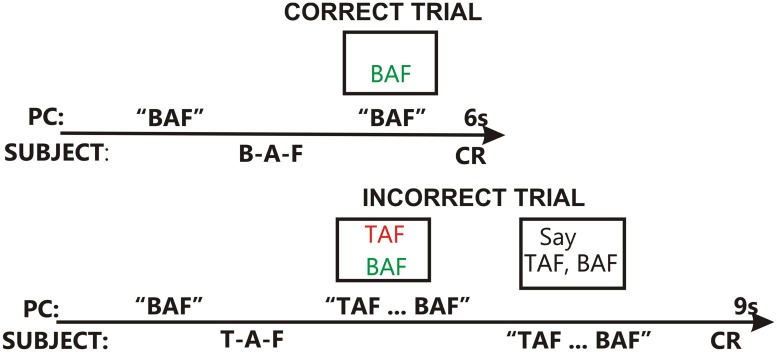
The structure of training trials. Top: Correct trial. After a correct response, the correctly detected token is shown in green font and the other exemplar of that token is played in quiet. Bottom: Incorrect trial. The incorrect and correct responses are shown in red and green fonts, respectively, and the two tokens are played in sequence in quiet. The listener articulates both syllables, emphasizing their phonetic differences.

The trial structure used in training is shown in Fig. 2. On each trial, the listener was presented with a CVC in speech-spectrum noise that spectrally matched the talker’s voice. SNR levels dynamically adjusted to provide appropriate masking levels for onset and coda consonants [53]. The listener typed a response on the keyboard. If the response was correct (Fig. 2, top), the correct CVC was shown in green font and the listener was presented with an articulation of the same CVC spoken by a different talker. This feedback syllable was presented without masking noise at 75 dB SPL. If the response was incorrect (Fig. 2, bottom), the syllable reported was shown in red font, and the correct syllable was shown in green font. Then, exemplars of both the correct syllable and incorrect response were presented in quiet. The listener was then instructed to articulate both tokens in a manner that exaggerated their phonetic contrasts.

CVCs were presented in blocks of 60 trials spoken by a single talker. Listeners were provided with percent correct scores after each 60-trial block. They were also notified of the overall difficulty level of each training session (i.e., the mean SNR). At the end of each training session, listeners were informed of their new SNR, and told when it exceeded their previous “personal best”.

All data were encrypted and automatically uploaded to a secure server after each training session. The uploaded data included a record of the time of occurrence and identity of each stimulus and response. In addition, the program calculated d’ values for each onset and coda consonant. The time and duration of training was also reported. If a listener failed to train for five successive days without a previously announced excuse (e.g., a planned vacation), they were contacted by telephone to assure that no technical problems had occurred. Listeners were encouraged to refrain from training if they were ill or experienced problems with their HAs. All listeners completed 40 days of training in less than 11 weeks.

Over the 40 days (20 sessions) of training, listeners identified 28,800 consonants in 14,400 randomly selected tokens. Because tokens were pseudo-randomly selected from the large training corpus (9,600 tokens), some individual tokens were never presented, whereas other tokens were presented multiple times over the two months of training. Each token was presented with two randomly selected, talker-specific, speech-spectrum noise samples; one in the left speaker and another in the right speaker. The delay between masker and syllable onset was also randomized on each trial. Hence, no two presentations of the same token were identical: each presentation was a unique combination of CVC, talker, syllable, exemplar, SNR, delay between masker onset and syllable onset, and noise samples in each channel.

### Statistical analysis

The data were analyzed with analysis of variance (ANOVA) for multifactorial repeated measures using the open-source CLEAVE program (T. J. Herron, www.ebire.org/hcnlab), which includes power analysis. The original degrees of freedom are reported for each test, with the significance levels adjusted using the Box/Greenhouse-Geisser correction for inhomogeneity of variance when appropriate [56]. ANOVAs were supplemented with Pearson correlation analyses [57] in order to explore the relationships between training, audiometric thresholds, and hearing aid benefit, and to explore the relationship between training-related improvements in consonant identification and improvements in SeRTs.

## Results

### Training impact on consonant identification thresholds

**Fig 3:**
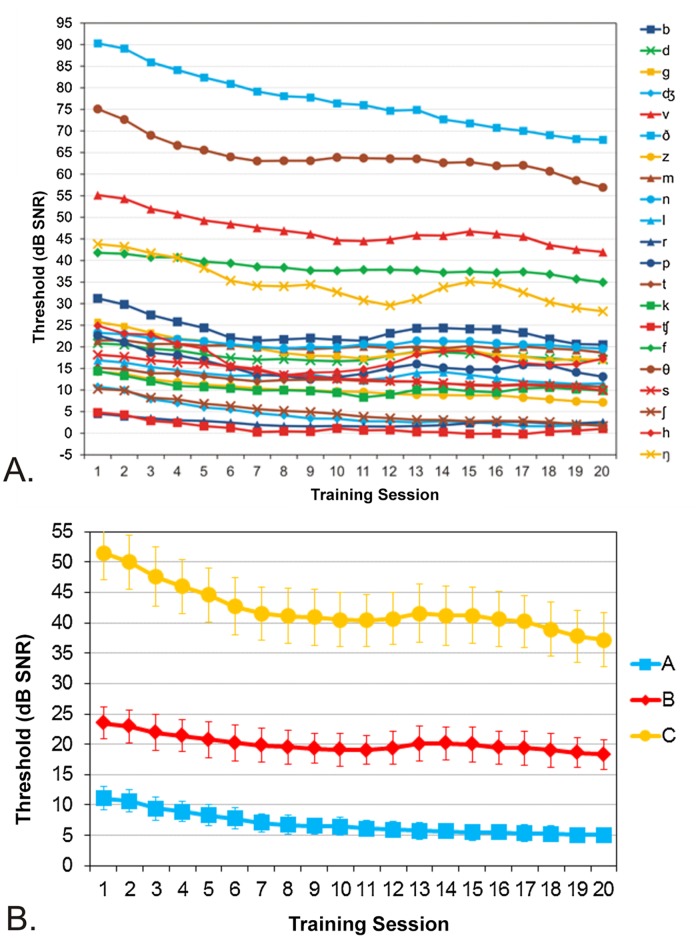
Mean consonant thresholds across training session. Top: individual consonants. Bottom: changes in mean consonant thresholds for consonant Groups A, B, and C during training. Error bars show standard errors that reflect inter-listener differences in threshold.

Fig. 3a shows the mean identification thresholds for each of the 21 consonants over the 20 in-home training sessions. Over two months, the consonant-identification thresholds used during training gradually improved by an average 8.2 dB SNR. Training benefits varied significantly across listeners, ranging from 2.8 dB to 22.3 dB. All consonant thresholds improved with training as shown in S1 Table, with the majority of consonants showing highly significant (p < 0.0001) improvements. Benefits occurred more rapidly in initial training sessions, but continued throughout training. Fig. 3b shows the average in-home improvements in thresholds for Group A, B, and C consonants. While training benefits were larger for Group C consonants than for Group A or Group B consonants, highly significant improvements (p< 0.0001) were seen for all consonant groups (S1 Table, bottom).

### Training effects on in-laboratory testing

**Fig 4:**
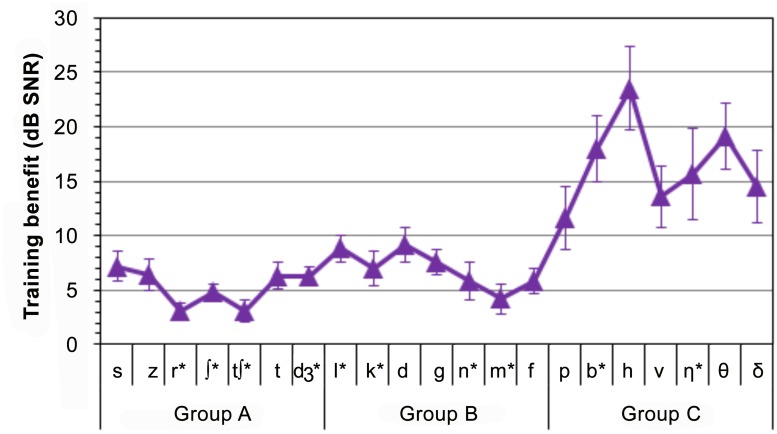
Training benefit on individual consonant identification thresholds. Measured in the laboratory before and after training. Error bars show standard errors. After training mean thresholds of consonants marked with asterisks were within the range seen in 16 ONH listeners.

Fig. 4 shows the mean training benefit (in dB SNR) for the different consonants, measured before and after training. Consonants are ordered as a function of intrinsic identification difficulty in young, normal-hearing (YNH) listeners, and divided into Groups A, B, and C [52,53]. Training benefits for different consonants ranged from 3.0 dB (/r/ and /tʃ/) to 26.5 dB (/h/), with a mean improvement of 9.1 dB (standard deviation = 4.8 dB).

ANOVAs examining the effects of training on the thresholds of individual consonants measured in the laboratory are provided in S2 Table. Training significantly improved in-laboratory performance for all consonants (*p* < 0.05), with all but /ʧ/ and /m/ showing performance improvements at a very high level of significance (*p* < 0.005). Consistent with the differences in training slopes shown in S1 Table, larger performance improvements were seen for the harder-to-identify Group C consonants (16.1 dB) than for the consonants in Group A (5.3 dB) or Group B (7.1 dB).

Training effects were further analyzed for the 19 consonants that occurred in both onset and coda position, with Training, Consonant, and Position as factors. Training produced a highly significant main effect [F(1,15) = 59.68, p < 0.0001, ω^2^ = 0.80]. Power analysis showed a 99% probability of detecting a training effect at a p< 0.05 significance level with a population of six OHI listeners. The Consonant main effect was also highly significant [F(18,270) = 121.55, p < 0.0001, ω^2^ = 0.89], with mean consonant thresholds varying from 2.3 dB for /ʧ/, to 74.9 dB for /ð/. There was also a Training x Consonant interaction [F(18,270) = 7.38, p < 0.0001, ω^2^ = 0.30] that reflected greater improvements for some consonants than others.

Training-related improvements were highly correlated across different consonant Groups [range r = 0.55 to r = 0.77, t(14) = 2.46 to t(14) = 4.52, p < 0.02 for all comparisons]. Total consonant training benefit was most strongly correlated with improvement in Group C consonant thresholds [r = 0.94]. A lower, but still significant correlation was seen between improvements in mean consonant-identification thresholds and improvement in SeRTs [r = 0.48, t(14) = 2.05, p < 0.03], and is discussed below.

**Fig 5:**
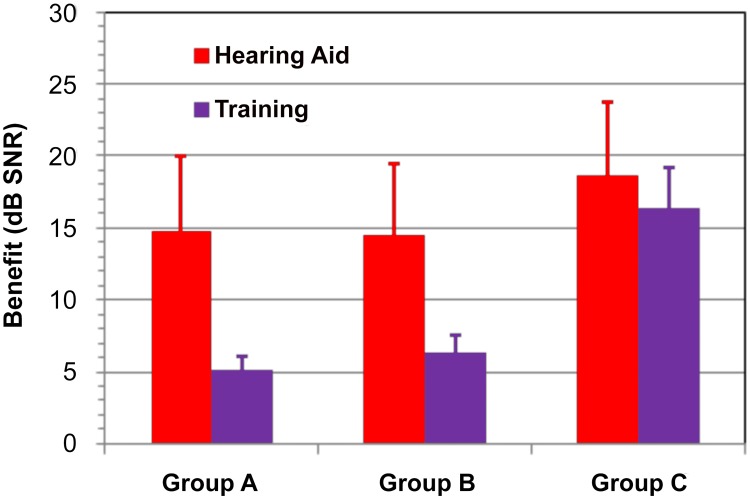
Hearing aid and training benefit for different consonant groups. Data from 11 listeners who participated in both studies. Error bars show standard errors.

Fig. 5 shows a comparison of HA benefit (aided vs. unaided listening) and training benefit for Group A, Group B, and Group C consonants in the 11 listeners tested in unaided and aided conditions prior to training. Relative to the magnitude of HA benefit, training improved Group A consonant thresholds by an additional 36%, Group B consonant thresholds by an additional 47%, and Group C consonant thresholds by an additional 94%.

**Fig 6:**
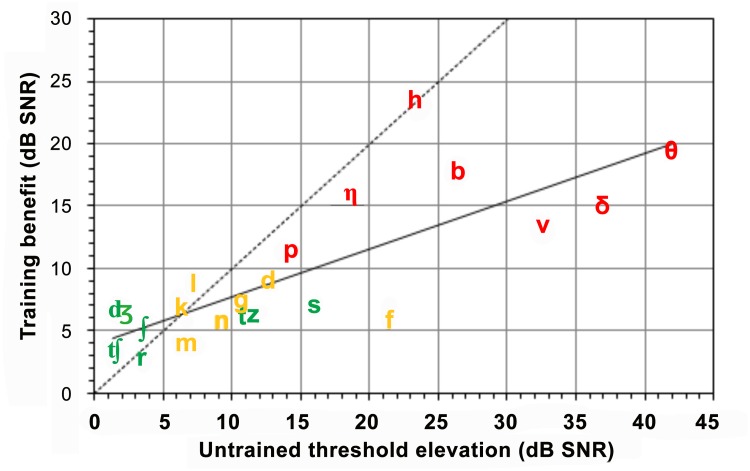
Training benefit as a function of untrained threshold elevation. Groups A, B and C consonants are plotted in green, yellow, and red, respectively. The diagonal dashed shows levels needed to restore performance to the average level of ONH listeners. The solid line shows the linear fit to the observed results.

Fig. 6 plots the magnitude of training benefit versus pre-training threshold elevations for different consonants. Overall, the consonants with greater pre-training threshold elevations showed greater training benefit [r = 0.78, t(14) = 4.66, p<0.001]. However, some frequently occurring Group A consonants (e.g., in green, /t/, /z/, and /s/) showed relatively small training benefits, despite relatively large pre-training threshold elevations.

Post-training thresholds remained elevated compared to ONH listeners [55] by an average of 2.0 dB for Group A consonants, 4.1 dB for Group B consonants, and 13.8 dB for Group C consonants. However, after training, seven consonants (marked with asterisks in Fig. 4) had mean thresholds that fell within the range of thresholds seen in 16 ONH listeners. These seven consonants included three from Group A (/ʃ/ and the affricates /ʧ/ and /ʤ/), two from Group B (/l/ and /k/), and two from Group C (/h/ and /ŋ/).

**Fig 7:**
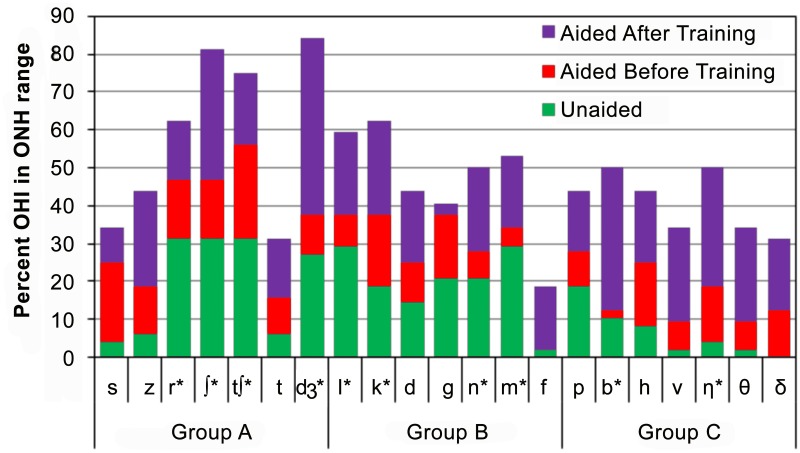
The percentage of OHI listeners whose thresholds were within the range of 16 ONH listeners. OHI data are shown for unaided listening, aided listening, and aided listening after training. Asterisks mark consonants where at least 50% OHI were in the range of ONH listeners after training.

Fig. 7 shows this percentage of listeners with consonant thresholds within the range observed in 16 ONH listeners [55] for each consonant in (1) Unaided listening (green), (2) Aided listening before training (red), and (3) Aided listening after training (purple). In unaided listening conditions, only a small percentage of listeners (e.g., 25–30%) had identification thresholds for any consonant within the normal range. With hearing aids, more consonant-identification thresholds fell within the normal range including three consonants in Group A (/r/, /ʃ/ and /ʧ/) whose thresholds fell within the normal range for more than 40% of listeners. Training produced further improvement for all consonants, so that 17 of 21 consonants were now within the normal range for more than 40% of listeners. Thus, while improvements due to training were smaller in dB terms than the improvements due to amplification, training resulted in greater numbers of consonants moving into the normal range of consonant-identification thresholds than did amplification. In other words, the thresholds for selected Group A and Group B consonants moved close to the normal range with amplification for many OHI listeners, and improved further into the normal range as a result of training.

### Training benefit on phonetic errors

**Fig 8:**
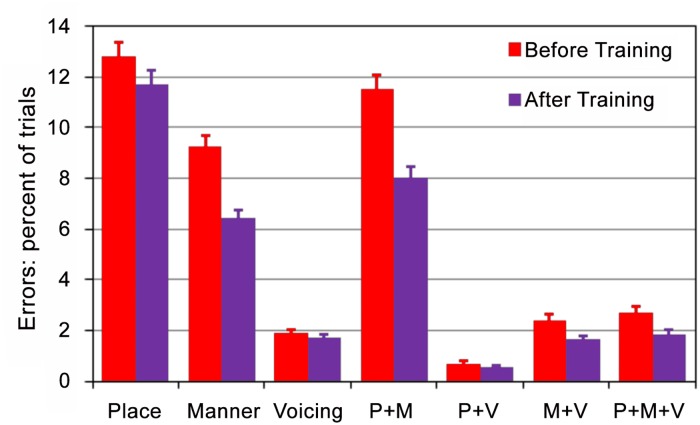
Training benefit on different types of phonetic errors. Error bars show standard errors.

Fig. 8 shows the percentage of trials with different types of phonetic errors before and after training. Although training reduced phonetic errors of all types, smaller reductions were seen for Place (-1.2%) and Voicing (-0.2%) errors than in with Manner (-3.2%) and Place + Manner (-4.9%) errors. Relatively large reductions were also seen in Manner + Voicing and Place + Manner + Voicing errors, although these errors were relatively infrequent in both pre- and post-training tests. As a result, the post-training pattern of phonetic errors more closely resembled the pattern of phonetic errors seen in YNH [53] and ONH listeners [55]; i.e., with a preponderance of place errors in comparison to errors of other types.

### Training benefit on consonant confusions

**Fig 9:**
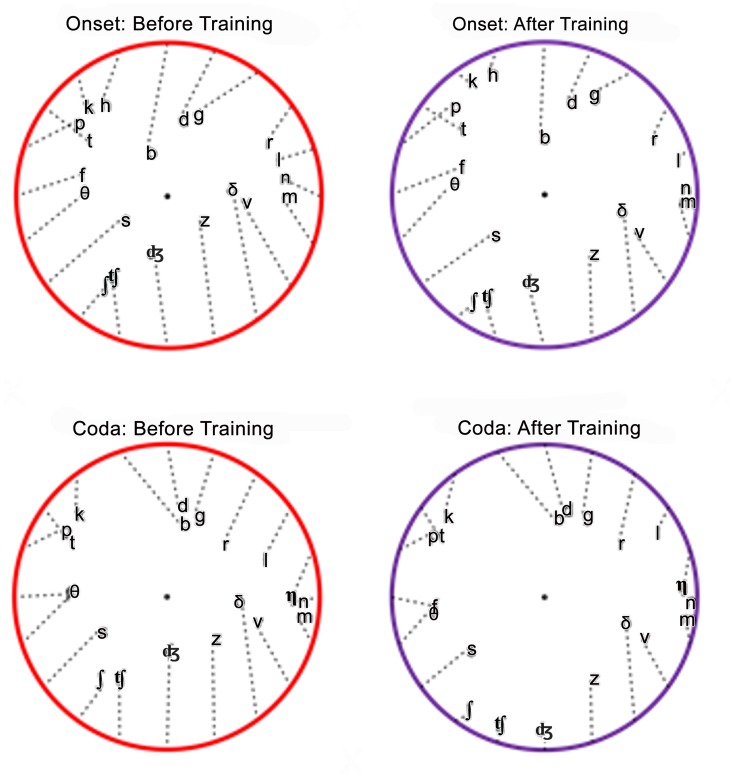
Barycentric consonant confusions circles before (left) and after (right) training. The incidence of consonant confusions is reflected in the distance between consonant pairs.

Fig. 9 shows the patterns of consonant confusions displayed using the barycentric clustering algorithm [53,55]. Consonants were initially placed in equidistant positions around a unit circle based on Voicing, Manner, and Place features using an optimized *a priori* consonant ordering. Then, the location of each consonant was modified based on the average of its initial position, weighted by its hit rate, and the position of every other consonant, weighted by the frequency of false responses to that consonant. Two movement iterations were used to generate the cluster plots shown. As a result of these iterations, each consonant was displaced from its initial position towards the locations of consonants with which it was confused (dotted lines in Fig. 9). The onset and coda consonant confusion matrices before training are provided in S3 and S4 Tables, and those after training are provided in S5 and S6 Tables.

The change between pre- and post-training barycentric plots can be characterized by the reestablishment of more normal consonant clustering due to reductions in manner and multi-feature errors. This resulted in post-training consonant locations that were closer to the circumference of the circle (e.g., /ʤ/ and /z/). Reduced confusions among certain consonant pairs (e.g., /ʃ/ and /ʧ/ and /h/ and /t/) also resulted in consonants moving closer to their original positions. In other cases, where performance improved but specific consonant pairs were still frequently confused with each other, the pair moved toward the circumference of the circle, but the consonants within the pair remained closely clustered, as seen for the nasals /m/ and /n/ and the fricatives /v/ and /ð/.

### Influences of talker familiarity, gender, SNR, and vowel

S1 Fig. shows consonant-identification performance at different SNRs before and after training for the tokens that were used in training (“trained voices”) and for the tokens spoken by unfamiliar talkers (“novel voices). Mean training-related improvements at the d’ = 2.2 performance level (approximately 66% correct) were 9–10 dB for the trained voices and 7–8 dB for the untrained voices. The Familiarity main effect was highly significant [F(1,15) = 35.77, p < 0.0001, ω^2^ = 0.70] due to better consonant identification in CVCs spoken by familiar talkers (63.3%) in comparison to CVCs spoken by unfamiliar talkers (60.8%). There was also a small but significant Training x Familiarity interaction [F(1,15) = 8.67, p < 0.02, ω^2^ = 0.34], reflecting larger improvements for tokens spoken by familiar (11.5%) than unfamiliar (9.0%) talkers. Although the training-related improvements were greater for tokens used in training, a supplementary ANOVA showed that training benefits remained highly significant when tested with syllables spoken by unfamiliar talkers [F(1,15) = 54.2, p < 0.0001, ω^2^ = 0.78]. Power analysis showed a 99% probability of detecting a significant effect at the p < 0.05 level in a group of seven OHI listeners.

To further analyze differences among talkers, vowels, and SNRs, the data were collapsed across consonants. We first analyzed the percentage of correct consonant detections with Training (before and after), Consonant position (onset and coda), Talker-gender (male vs. female), SNR (B-6, B, and B+6), and Familiarity (trained vs. novel talkers) as factors. Training produced a highly significant mean improvement of 10.2% in consonant-identification performance [F(1,15) = 60.24, p < 0.0001, ω^2^ = 0.80]. Consonant-position effects failed to reach significance [F(1,15) = 2.91, p < 0.15], and there was no significant Training x Consonant-Position interaction [F(1,15) = 0.31, NS]. Talker-gender effects also failed to reach significance [F(1,15) = 0.06, NS], without a significant Training x Talker-Gender interaction [F(1,15) = 0.57, NS]. SNR had a predictably large effect [F(2, 30) = 137.62, p < 0.0001, ω^2^ = 0.90], with performance improving by 16.6% over the 12 dB SNR range. However, training benefits did not differ significantly as a function of SNR [F(2,30) = 1.89, NS].

As expected by the lack of Training x SNR interaction, training did not result in a significant change in psychometric slopes [mean 1.38%/dB, F(1,15) = 2.56, p < 0.15], which remained much shallower in OHI listeners than in ONH listeners [7]. We also analyzed the effects of training on syllable intensity effects by comparing the percentage of consonants correctly identified in high-intensity syllables (73–75 dB SPL) versus low-intensity syllables (70–72 dB SPL). This analysis revealed a significant effect of syllable intensity [2.9%, F(1,15) = 39.3, p < 0.0001, ω^2^ = 0.72] that was not significantly altered by training [F(1,23) = 0.29, NS].

Training effects on syllables containing different vowels were analyzed in another ANOVA with Training, Position, Vowel, and SNR as factors. As reported previously [7], we found that aided OHI listeners had more difficulty in identifying consonants in syllables containing /i/ than in syllables containing /ɑ/ or /u/ [F(2,30) = 13.04, p< 0.0002, ω^2^ = 0.18]. Training effects did not differ for syllables containing different vowels [F(2,30) = 1.12, NS], and there was no significant Training x Vowel x Position interaction [F(2,30) = 1.09, NS].

### Inter-listener differences in training benefit

Table 1 shows pure-tone average thresholds (PTA, 500, 1000, and 2000 Hz), training benefits for Group A, B, and C consonants, mean benefit and benefit z-scores (relative to ONH listeners), and SeRT benefits and z-scores (discussed below). Consonant-identification performance improved in every listener (range 3.7 to 19.8 dB), with 94% showing significant improvements (i.e., z-score changes > 2.0). Surprisingly, the magnitude of training benefit was not significantly correlated with pre-training performance [r = 0.06, NS]. For example, among the five listeners with the highest pre-training consonant-identification thresholds, training improvements ranged from 4 to 18 dB, whereas among the five listeners with the lowest pre-training thresholds, improvements ranged from 4 to 14 dB.

**Table 1:** Training results from individual listeners.

Listener	PTA	Group A benefit	Group B benefit	Group C benefit	Mean benefit	Mean z-score	SeRT Benefit	SeRT z-score
1	24.17	1.49	0.97	13.00	5.15	2.71	-0.06	-0.63
2	36.67	4.63	2.24	5.21	4.02	2.12	-1.21	-1.10
3	49.17	6.20	9.15	15.51	10.28	5.41	3.52	3.20
4	29.17	1.44	5.33	9.77	5.51	2.90	-1.56	-1.42
5	30.00	11.90	15.08	27.34	18.11	9.53	-0.03	-0.03
6	32.50	2.86	5.37	1.89	3.37	1.77	0.35	0.32
7	21.67	5.38	5.75	23.06	11.40	6.00	1.70	1.54
8	32.50	6.63	9.24	15.98	10.62	5.59	1.02	-0.56
9	44.17	6.45	7.91	8.66	7.67	4.04	0.45	-0.27
10	10.00	3.46	6.04	29.67	13.06	6.87	0.69	0.63
11	22.50	2.28	6.32	10.54	6.38	3.36	0.21	0.19
12	45.00	2.17	3.07	13.82	6.35	3.34	-0.56	-0.51
13	13.33	5.21	11.82	25.15	14.06	7.40	0.26	0.24
14	44.17	5.46	2.63	3.61	3.90	2.05	-0.33	-0.30
15	14.17	10.77	11.10	31.14	17.67	9.30	1.76	1.60
16	46.67	8.68	8.73	22.73	13.38	7.04	1.23	1.12
**MEAN**	**31.77**	**5.31**	**6.92**	**16.07**	**9.43**	**4.97**	**0.46**	**0.25**

Data from the 16 listeners who underwent training including pure-tone average thresholds (PTA), training benefit in dB SNR for different consonant groups, mean benefit averaged over consonants, and z-score measures of improvement (positive values indicate improvement), training effects on sentence reception thresholds (SeRTs) and corresponding SeRT z-score changes.

To further understand the factors predicting training benefit, we first analyzed the correlation between audiometric thresholds and training benefit. There was an insignificant negative correlation between overall improvement and the PTA [r = -0.39, t(14) = 1.59, p < 0.15]. There was also no significant correlation of mean training benefit with trainee age [r = 0.09, t(14) = 0.34, NS].

### Training benefit on sentence processing

Training produced small mean SeRT improvements (0.46 dB) that failed to reach significance either for the HINT [0.79 dB, F(1,15) = 2.49, p < 0.12] or the QSIN [0.14 dB, F(1,15) = 0.15, NS]. Only one listener showed a significant training-related improvement in SeRTs (see Table 1). However, listeners who showed more consonant-identification training benefit showed greater improvements in SeRTs [r = 0.48, t(14) = 2.05, p < 0.03].

## Discussion

Two months of consonant-identification training enabled OHI listeners to substantially improve their consonant-identification thresholds during training sessions. Highly significant, 9.1 dB improvements were also seen in laboratory tests, with 94% of trained listeners showing significant training benefit. The improvements were equivalent for onset and coda consonants, syllables spoken by male and female talkers, syllables presented at different SNRs, and syllables with different vowel nuclei. Although improvements were larger for tokens used in training, 78% of the training benefit generalized to syllables spoken by unfamiliar talkers.

Training reduced consonant-identification deficits for all consonants and enabled more than 40% of OHI listeners to identify most consonants at SNRs within the range of ONH listeners. Moreover, the patterns of consonant confusions normalized, reducing multi-feature errors and increasing the relative incidence of place-only errors to more closely approximate the pattern seen in normal-hearing listeners. This indicated that more consonant information was extracted after training even for incorrectly identified syllables.

Importantly, these improvements were seen during training using a very large training set (1200 CVCs and 9,600 tokens). Because the occurrence of each consonant was statistically independent from that of other phonemes, users could not alter their response bias based on partial syllable information; i.e., the accurate identification of one consonant and vowel in a syllable provided no predictive information about the identity of the other consonant. Hence, the results demonstrate perceptual learning of consonant discriminations in a manner that would be expected to generalize to other speech contexts. This contrasts with training protocols using more limited and statistically dependent stimulus and response sets [25,26,30]. For example, in Humes’s training protocol [25], listeners in some sessions were trained with 50 token words of 1,2, or 3 syllable length, and selected the token presented from an alphabetized list on a computer monitor. As listeners became familiar with the training set, identifying the number of syllables in a token would reduce the number of possible foils, and additional information about vowels and easily-identified consonants in the token would often enable the listener to determine which token had been presented, even if the more difficult consonants in the token remained indiscriminate. Thus, improvements seen with such closed-set training paradigms would reflect the statistical dependencies of the training set, and be unlikely to generalize to other spoken material.

### Other possible explanations for training improvements

Training produced gradual improvements in the identification thresholds of all consonants. One possible explanation is procedural learning. For example, listeners might have become more familiar with alphabetical symbols used to code consonants (e.g., /DH/ for /ð/). However, while such incidental learning might contribute to performance improvements in initial training sessions, it would not explain the continued improvements in later training sessions, or the improved performance observed in laboratory testing when spoken responses, rather than typed responses, were obtained.

Content learning also appears unlikely to have played a significant role in the improvements observed. Of course, listeners would learn to limit their responses to consonants drawn from a set of 20 onset and 20 coda consonants over the course of training, and hence would became biased to report these consonants. For example, if tested with a syllable containing a consonant not included in the training set (e.g., /wal/), listeners might be biased to report a trained syllable (e.g., /lal/). However, since the consonant set included almost all common consonants in spoken American English, any biases acquired during content learning would be expected to have a relatively small effects. Similarly, content learning of idiosyncratic articulations of particular tokens could not contribute to improved performance; because of the large size of the randomly-sampled token set (9,600 tokens), many individual tokens were presented only once or not at all during the two months of training.

The current paradigm can be contrasted with that of our previous study [37], where OHI listeners learned to identify nine unvoiced onset consonants in CV syllables and nine voiced coda in VC syllables. In that study, training-related alterations in response bias would be expected to reduce the incidence of reporting voiced consonants at syllable onset (e.g., /bal/ would be reported as /pal/) and unvoiced consonants in syllable coda (e.g., /rak/ would be reported as /rad/).

Although training improved identification accuracy in both the current and previous training study by roughly 10%, part of the performance improvement in our previous study [37] may have reflected more subtle alterations in response bias. Both the onset and coda consonant sets used in that study contained consonants with identification thresholds differing by more than 30 dB (e.g., /ʧ/ vs. /ɵ/ and /m/ vs. /ð/). Because only two SNRs were used in testing (0 and 10 dB), OHI listeners would have been able to identify some consonants (e.g., /ʧ/) much more easily than others (e.g. /ɵ/). With training, listeners likely learned which consonants could not be identified, and adjusted their response bias accordingly, e.g., guessing “/ɵ/” when no onset consonant could be clearly identified. Such a change in guessing strategy would have had little influence on the results of the current experiment because each consonant was presented at three different SNRs, centered on an SNR designed to equate the identifiability of different consonants.

What did the OHI listeners learn throughout the training process? Earlier, we hypothesized that some of the deficits in consonant identification seen in aided OHI listeners were due to suboptimal utilization of high-frequency consonant cues. Even with HAs, the OHI listeners had difficulty in detecting short-duration plosive bursts and high-frequency frication. Our results suggest that some of these cues were audible in aided listening conditions, but were not being effectively utilized. After training, thresholds improved by more than 10 dB for /b/, /p/, /h/, /ŋ/, /ɵ/, /v/, and /ð/ as aided OHI listeners improved their utilization of burst and frication cues.

Although training significantly improved consonant-identification performance and restored the thresholds of some consonants to near-normal levels, the post-training performance of OHI listeners continued to differ in two important respects from the performance of ONH listeners. First, the psychometric slopes of trained OHI listeners remained much shallower than those of ONH listeners, and did not improve with training. This suggests that performance was limited by distortion factors such as impaired frequency resolution [58] and altered temporal processing [59]. Second, the performance of OHI listeners remained influenced by syllable intensity (independent of SNR) after training, suggesting that post-training performance remained limited by consonant inaudibility [60].

### Limited training benefit on SeRTs

The SeRTs of normal hearing listeners are below their mean consonant-identification thresholds, and these differences are substantially increased in OHI listeners [7,52,55]. This indicates that OHI listeners identify only a subset of consonants in the sentences used in SeRT testing. In the HINT and QSIN, the consonants identified are likely those that occur in stressed words early in sentences, where SNRs are 8–10 dB higher than for the words that occur later in the sentence [52]. Nevertheless, consonant-identification thresholds accurately predict HINT and QSIN SeRTs, presumably because some consonants must be identified in order for a sentence to be understood. In a previous study, we examined speech comprehension in 24 OHI listeners, including 11 of the listeners included in the current study [7]. Unaided SeRTs were accurately predicted by Group A consonant thresholds, and HA benefits on SeRTs (2.0 dB) were accurately predicted by the HA benefit (15.5 dB) on Group A consonants. However, only about 13% of the HA benefit on Group A consonants generalized to SeRTs.

The limited generalization of HA benefit from consonants to sentences reflects in part the fact that the SeRTs of aided OHI listeners show much smaller elevations than consonant-identification thresholds [7]. This suggests that OHI listeners are able to understand the sentences of the HINT and QSIN with minimal consonant information, supplemented by vowel and intonation cues, accurate determination of syllabic structure, and top-down semantic processing. Top-down processing is particularly facilitated in HINT and QSIN sentences because they have highly constrained syllable counts, word length, vocabulary, syntax, and semantic structure. For example, the length of HINT sentences ranges from 6 to 8 syllables, with all sentences having a simple declarative structure (e.g., “The cat caught a little mouse”). Word selection is also highly constrained: 22% of sentences contain only monosyllabic words, and 52% of sentences contain a single 2-syllable word. Three-syllable words occur in less than 12% of sentences. Word choice is similarly constrained: only commonly occurring words at a 1^st^ grade reading level are used. Word choice in QSIN sentences is also constrained, but sentences show a greater range in length (7–12 syllables) and syntactic structure. However, the syllable counts of QSIN words are more restricted than those of the HINT: 91.3% of sentences contain a single 2-syllable word, and 3-syllable words never occur. As a result of these lexical, syllabic, syntactic, and semantic constraints, OHI listeners can understand sentences at SNRs far below those needed to identify consonants in isolated syllables. Of course, top-down processing exerts its greatest influence in highly constrained sentences where OHI listeners perform at near normal levels [61], but comprehension declines substantially in OHI listeners as listening materials increase in complexity [62,63].

In the current study, training produced relatively small improvements in Group A consonant thresholds (4.13 dB) for syllables spoken by unfamiliar talkers. Assuming that this improvement generalized to SeRTs to the same degree as HA benefit (i.e., 13%), the training-related improvement predicted in SeRTs would be quite small (0.54 dB) and similar to the observed training benefit (0.42 dB). However, as with SeRT benefits seen with HAs, training-related improvements in consonant-identification thresholds correlated significantly with training improvements in SeRTs. Thus, consonant-identification training may have produced small benefits even for HINT and QSIN sentences.

In contrast to HAs, which produce relatively similar benefits for Groups A, B, and C consonants, training produced larger benefits for less-easily identified Group B, and particularly Group C consonants. What speech-comprehension benefit might result from the improved identification of these consonants? An analysis of the SUBTLEX film dialog database [40] shows that Group B and Group C consonants constitute more than 60% of all consonants used in speech. However, the identification thresholds of these consonants are far above SeRTs measured with the HINT and QSIN, suggesting that their identification contributes little to the perception of HINT and QSIN sentences. Of course, the accurate identification of these consonants may play a more significant role in understanding more complex listening material. This implies that training-related improvements in identifying more difficult consonants would improve the understanding of sentences expressing more complex ideas, with less predictable grammatical structures and vocabulary. Thus, reduced semantic and grammatical constraints would force listeners to rely to a greater extent on bottom-up consonant information. However, no sentence tests are available to test this hypothesis. Identifying a larger proportion of consonants should also reduce the cognitive load of processing more complex speech, but standardized tests of cognitive load during speech comprehension have not yet been developed.

## Conclusions

Hearing loss impairs the ability of OHI listeners to identify phonemes, particularly consonants, by degrading the acoustic cues available for understanding conversational speech. One of the central goals of hearing aid prescription is to restore the acoustic cues needed for accurate phoneme processing. However, hearing aids alone are insufficient to restore normal consonant identification in the majority of OHI listeners [7]. As a result, recent research programs have focused on the potential benefits of perceptual training [13,64].

What is the appropriate target of training? Considerable evidence suggests that speech-comprehension deficits of OHI listeners reflect difficulty in identifying consonants [1,7,9]. The current experiment extended our previous studies of consonant identification training [37] by carefully controlling for a variety of potentially confounding factors and using signal detection measures to separate improvements in sensory processing (d’) from alterations in response criteria (β). We found that consonant-identification training resulted in large and consistent improvements in consonant-identification thresholds for virtually all OHI listeners and for all consonants. Moreover, these improvements generalized to tokens spoken by unfamiliar talkers. Thus, perceptual training enabled experienced HA users to improve their listening performance, restoring their ability to identify some consonants into the normal range. Thus, remediating consonant-identification deficits through perceptual training is a promising approach to audiological rehabilitation [28]. However, although improvements were seen for virtually all consonants, they were larger for the more difficult-to-identify Group B and Group C consonants. The accurate identification of these consonants has little influence on the SeRTs measured with the simple sentence tests. As a result, training produce limited improvement in SeRTs measured with the HINT and QSIN. However, Group B and Group C consonants constitute more than 60% of consonant occurrences in spoken English, and are therefore likely to play a more critical role in understanding speech in listening conditions that are more semantically and syntactically complex than the simple sentences of the HINT and QSIN.

## Supporting Information

S1 Fig Performance before and after training for familiar and unfamiliar voices. Consonant-identification performance before (red) and after (purple) training for tokens used in training (solid lines) and for tokens spoken by unfamiliar talkers (dashed lines). Percent correct scores are shown at three SNRs, relative to listener-specific and consonant-specific baseline (B). Error bars show standard errors.
(TIF)

S1 Table Training improvements in individual consonants across the training sessions. Shown are the slopes of the linear regression across training sessions and the correlation of consonant-identification thresholds with training sessions for individual consonants and consonant groups.
(DOCX)

S2 Table Consonant-identification thresholds before and after training. Consonant-identification thresholds (dB SNR) before and after training as measured in the laboratory, ANOVA analysis of training effects, and significance of improvement for individual consonants and consonant groups.
(DOCX)

S3 Table Confusion matrix for onset consonants before training. Each row gives the number of consonant responses of each type for the consonant at the top of the column.
(DOCX)

S4 Table Confusion matrix for coda consonants before training. Each row gives the number of consonant responses of each type for the consonant at the top of the column.
(DOCX)

S5 Table Confusion matrix for onset consonants after training. Each row gives the number of consonant responses of each type for the consonant at the top of the column.
(DOCX)

S6 Table Confusion matrix for coda consonants after training. Each row gives the number of consonant responses of each type for the consonant at the top of the column.
(DOCX)

## References

[1] Kuk F, Lau CC, Korhonen P, Crose B, Peeters H, et al (2010) Development of the ORCA nonsense syllable test. Ear Hear 31: 779–795. 10.1097/AUD.0b013e3181e97bfb 20622673

[2] Schwartz DM, Surr RK (1979) Three experiments on the California Consonant Test. J Speech Hear Disord 44: 61–72. 423556 10.1044/jshd.4401.61

[3] Dubno JR, Dirks DD, Schaefer AB (1989) Stop-consonant recognition for normal-hearing listeners and listeners with high-frequency hearing loss. II: Articulation index predictions. J Acoust Soc Am 85: 355–364. 2921418 10.1121/1.397687

[4] Dubno JR, Dirks DD, Ellison DE (1989) Stop-consonant recognition for normal-hearing listeners and listeners with high-frequency hearing loss. I: The contribution of selected frequency regions. J Acoust Soc Am 85: 347–354. 2921417 10.1121/1.397686

[5] Gordon-Salant S (1987) Consonant recognition and confusion patterns among elderly hearing-impaired subjects. Ear Hear 8: 270–276. 3678640 10.1097/00003446-198710000-00003

[6] Marriage JE, Moore BC (2003) New speech tests reveal benefit of wide-dynamic-range, fast-acting compression for consonant discrimination in children with moderate-to-profound hearing loss. Int J Audiol 42: 418–425. 14582638 10.3109/14992020309080051

[7] Woods DL, Arbogast TL, Doss Z, Younus M, Herron TJ, et al (2014) Aided and unaided speech perception by older hearing impaired listeners PLoS ONE. In Press. 10.1371/journal.pone.0114922 PMC4346396 25730423

[8] Saripella R, Loizou PC, Thibodeau L, Alford JA (2011) The effects of selective consonant amplification on sentence recognition in noise by hearing-impaired listeners. J Acoust Soc Am 130: 3028–3037. 10.1121/1.3641407 22087930 PMC3248061

[9] Phatak SA, Yoon YS, Gooler DM, Allen JB (2009) Consonant recognition loss in hearing impaired listeners. J Acoust Soc Am 126: 2683–2694. 10.1121/1.3238257 19894845 PMC2787079

[10] Strelcyk O, Li N, Rodriguez J, Kalluri S, Edwards B (2013) Multichannel compression hearing aids: effect of channel bandwidth on consonant and vowel identification by hearing-impaired listeners. J Acoust Soc Am 133: 1598–1606. 10.1121/1.4789894 23464029

[11] Ahlstrom JB, Horwitz AR, Dubno JR (2014) Spatial separation benefit for unaided and aided listening. Ear Hear 35: 72–85. 10.1097/AUD.0b013e3182a02274 24121648 PMC3872487

[12] Ahlstrom JB, Horwitz AR, Dubno JR (2009) Spatial benefit of bilateral hearing AIDS. Ear Hear 30: 203–218. 10.1097/AUD.0b013e31819769c1 19194292 PMC3693091

[13] Henshaw H, Ferguson MA (2013) Efficacy of individual computer-based auditory training for people with hearing loss: a systematic review of the evidence. PLoS One 8: e62836 10.1371/journal.pone.0062836 23675431 PMC3651281

[14] Sweetow RW, Sabes JH (2006) The need for and development of an adaptive Listening and Communication Enhancement (LACE) Program. J Am Acad Audiol 17: 538–558. 16999250 10.3766/jaaa.17.8.2

[15] Killion MC, Niquette PA, Gudmundsen GI, Revit LJ, Banerjee S (2004) Development of a quick speech-in-noise test for measuring signal-to-noise ratio loss in normal-hearing and hearing-impaired listeners. J Acoust Soc Am 116: 2395–2405. 15532670 10.1121/1.1784440

[16] Nilsson M, Soli SD, Sullivan JA (1994) Development of the Hearing in Noise Test for the measurement of speech reception thresholds in quiet and in noise. J Acoust Soc Am 95: 1085–1099. 8132902 10.1121/1.408469

[17] Olson AD, Preminger JE, Shinn JB (2013) The effect of LACE DVD training in new and experienced hearing aid users. J Am Acad Audiol 24: 214–230. 10.3766/jaaa.24.3.7 23506666

[18] Saunders GH, Teahen M (2010) Auditory Training for Veterans. NCRAR Seminar Series January 21.

[19] Bilger RC, Nuetzel JM, Rabinowitz WM, Rzeczkowski C (1984) Standardization of a test of speech perception in noise. J Speech Hear Res 27: 32–48. 6717005 10.1044/jshr.2701.32

[20] Fu QJ, Galvin JJ 3rd, Wang X, Nogaki G (2005) Moderate auditory training can improve speech performance of adult cochlear implant patients. Acoustics Research Letters Online 6: 106–111.

[21] Oba SI, Fu QJ, Galvin JJ 3rd (2011) Digit Training in Noise Can Improve Cochlear Implant Users’ Speech Understanding in Noise. Ear Hear. 10.1097/AUD.0b013e31820fc821 PMC3129451 21389857

[22] Burk MH, Humes LE, Amos NE, Strauser LE (2006) Effect of training on word-recognition performance in noise for young normal-hearing and older hearing-impaired listeners. Ear Hear 27: 263–278. 16672795 10.1097/01.aud.0000215980.21158.a2

[23] Burk MH, Humes LE (2007) Effects of training on speech recognition performance in noise using lexically hard words. J Speech Lang Hear Res 50: 25–40. 17344546 10.1044/1092-4388(2007/003)

[24] Burk MH, Humes LE (2008) Effects of long-term training on aided speech-recognition performance in noise in older adults. J Speech Lang Hear Res 51: 759–771. 10.1044/1092-4388(2008/054) 18506049 PMC3179269

[25] Humes LE, Burk MH, Strauser LE, Kinney DL (2009) Development and efficacy of a frequent-word auditory training protocol for older adults with impaired hearing. Ear Hear 30: 613–627. 10.1097/AUD.0b013e3181b00d90 19633564 PMC3210026

[26] Humes LE, Kinney DL, Brown SE, Kiener AL, Quigley TM (2014) The effects of dosage and duration of auditory training for older adults with hearing impairment. J Acoust Soc Am 136: EL224 10.1121/1.4890663 25190425 PMC4144170

[27] Kuchinsky SE, Ahlstrom JB, Cute SL, Humes LE, Dubno JR, et al (2014) Speech-perception training for older adults with hearing loss impacts word recognition and effort. Psychophysiology. 10.1111/psyp.12242 PMC4234634 24909603

[28] Miller JD, Watson CS, Kistler DJ, Wightman FL, Preminger JE (2008) Preliminary evaluation of the speech perception assessment and training system (SPATS) with hearing-aid and cochlear-implant users. Proc Meet Acoust 2: 1–9. 19305636 10.1121/1.2988004 PMC2658626

[29] Watson CS, Miller JD, Kewley-Port D, Humes LE, Wightman FL (2008) Training listeners to identify the sounds of speech: I. A review of past studies. Hear J 61: 26 20098630 10.1097/01.hj.0000339502.52055.d8 PMC2809377

[30] Miller JD, Watson CS, Kistler DJ, Preminger JE, Wark DJ (2008) Training listeners to identify the sounds of speech: II. Using SPATS software. Hear J 61: 29–33. 20209044 10.1097/01.HJ.0000341756.80813.e1 PMC2832483

[31] Dubno JR (2013) Benefits of auditory training for aided listening by older adults. Am J Audiol 22: 335–338. 10.1044/1059-0889(2013/12-0080) 23975126 PMC4051705

[32] Gold JR, Bajo VM (2014) Insult-induced adaptive plasticity of the auditory system. Front Neurosci 8: 110 10.3389/fnins.2014.00110 24904256 PMC4033160

[33] Alexander JM, Kluender KR (2009) Spectral tilt change in stop consonant perception by listeners with hearing impairment. J Speech Lang Hear Res 52: 653–670. 10.1044/1092-4388(2008/08-0038) 18952854 PMC2749884

[34] Coez A, Belin P, Bizaguet E, Ferrary E, Zilbovicius M, et al (2010) Hearing loss severity: impaired processing of formant transition duration. Neuropsychologia 48: 3057–3061. 10.1016/j.neuropsychologia.2010.06.016 20600193

[35] Revoile SG, Holden-Pitt L, Pickett JM (1985) Perceptual cues to the voiced-voiceless distinction of final fricatives for listeners with impaired or with normal hearing. J Acoust Soc Am 77: 1263–1265. 3980876 10.1121/1.392199

[36] Hedrick MS, Carney AE (1997) Effect of relative amplitude and formant transitions on perception of place of articulation by adult listeners with cochlear implants. J Speech Lang Hear Res 40: 1445–1457. 9430763 10.1044/jslhr.4006.1445

[37] Stecker GC, Bowman GA, Yund EW, Herron TJ, Roup CM, et al (2006) Perceptual training improves syllable identification in new and experienced hearing aid users. J Rehabil Res Dev 43: 537–552. 17123192 10.1682/jrrd.2005.11.0171

[38] Wilson RH, Bell TS, Koslowski JA (2003) Learning effects associated with repeated word-recognition measures using sentence materials. J Rehabil Res Dev 40: 329–336. 15074444 10.1682/jrrd.2003.07.0329

[39] Yund EW, Woods DL (2010) Content and procedural learning in repeated sentence tests of speech perception. Ear Hear 31: 769–778. 10.1097/AUD.0b013e3181e68e4a 20562624

[40] Brysbaert M, New B (2009) Moving beyond Kucera and Francis: a critical evaluation of current word frequency norms and the introduction of a new and improved word frequency measure for American English. Behav Res Methods 41: 977–990. 10.3758/BRM.41.4.977 19897807

[41] Brennan SE, Clark HH (1996) Conceptual pacts and lexical choice in conversation. J Exp Psychol Learn Mem Cogn 22: 1482–1493. 8921603 10.1037//0278-7393.22.6.1482

[42] Recanzone GH, Schreiner CE, Merzenich MM (1993) Plasticity in the frequency representation of primary auditory cortex following discrimination training in adult owl monkeys. Journal of Neuroscience 13: 87–103. 8423485 10.1523/JNEUROSCI.13-01-00087.1993 PMC6576321

[43] Whitton JP, Hancock KE, Polley DB (2014) Immersive audiomotor game play enhances neural and perceptual salience of weak signals in noise. Proc Natl Acad Sci U S A 111: E2606–2615. 10.1073/pnas.1322184111 24927596 PMC4078866

[44] Moreno S, Bidelman GM (2014) Examining neural plasticity and cognitive benefit through the unique lens of musical training. Hear Res 308: 84–97. 10.1016/j.heares.2013.09.012 24079993

[45] Ferguson MA, Henshaw H, Clark DP, Moore DR (2014) Benefits of phoneme discrimination training in a randomized controlled trial of 50- to 74-year-olds with mild hearing loss. Ear Hear 35: e110–121. 10.1097/AUD.0000000000000020 24752284 PMC4072445

[46] Ahissar M, Nahum M, Nelken I, Hochstein S (2009) Reverse hierarchies and sensory learning. Philos Trans R Soc Lond B Biol Sci 364: 285–299. 10.1098/rstb.2008.0253 18986968 PMC2674477

[47] Goldstone RL (1998) Perceptual learning. Annu Rev Psychol 49: 585–612. 9496632 10.1146/annurev.psych.49.1.585

[48] Bradlow AR, Akahane-Yamada R, Pisoni DB, Tohkura Y (1999) Training Japanese listeners to identify English /r/ and /l/: long-term retention of learning in perception and production. Percept Psychophys 61: 977–985. 10499009 10.3758/bf03206911 PMC3472521

[49] Piisoni DB (1997) Some Thoughts on “Normalization” in Speech Perception In: Joshnson KaM J. W., editor. Talker Variability in Speech Processing. San Diego, Ca: Academic Press pp. 9–32.

[50] Eckman F, Elreyes A, Iverson GK (2003) Some Principles of Second Language Phonology. Second Language Research 19: 169–208.

[51] Port R, Leary A (2005) Against formal phonology. Language 81: 927–964.

[52] Woods DL, Yund EW, Herron TJ (2010) Measuring consonant identification in nonsense syllables, words, and sentences. J Rehabil Res Dev 47: 243–260. 20665350 10.1682/jrrd.2009.04.0040

[53] Woods DL, Yund EW, Herron TJ, Cruadhlaoich MA (2010) Consonant identification in consonant-vowel-consonant syllables in speech-spectrum noise. J Acoust Soc Am 127: 1609–1623. 10.1121/1.3293005 20329860

[54] Boothroyd A (2010) Adapting to changed hearing: the potential role of formal training. J Am Acad Audiol 21: 601–611. 10.3766/jaaa.21.9.6 21241648

[55] Woods DL, Doss Z, Herron TJ, Yund EW (2013) Age-related changes in consonant and sentence processing. J Rehabil Res Dev 49: 1277–1291. 10.1682/jrrd.2011.08.0150 23341320

[56] Greenhouse SW, Geisser S (1959) On Methods in the Analysis of Profile Data Psychometrika 24: 95–112.

[57] Whittaker J (1990) Graphical Models in Applied Multivariate Statistics. Chichester, England: J. Wiley and Sons.

[58] Turner CW (2006) Hearing loss and the limits of amplification. Audiol Neurootol 11 Suppl 1: 2–5. 10.1159/000095606 17063003

[59] Reed CM, Braida LD, Zurek PM (2009) Review article: review of the literature on temporal resolution in listeners with cochlear hearing impairment: a critical assessment of the role of suprathreshold deficits. Trends Amplif 13: 4–43. 10.1177/1084713808325412 19074452 PMC2880464

[60] Humes LE (2007) The contributions of audibility and cognitive factors to the benefit provided by amplified speech to older adults. J Am Acad Audiol 18: 590–603. 18236646 10.3766/jaaa.18.7.6

[61] Cox RM, Gray GA, Alexander GC (2001) Evaluation of a Revised Speech in Noise (RSIN) test. J Am Acad Audiol 12: 423–432. 11599877

[62] Benichov J, Cox LC, Tun PA, Wingfield A (2012) Word recognition within a linguistic context: effects of age, hearing acuity, verbal ability, and cognitive function. Ear Hear 33: 250–256. 10.1097/AUD.0b013e31822f680f 21918453 PMC3253325

[63] Stewart R, Wingfield A (2009) Hearing loss and cognitive effort in older adults’ report accuracy for verbal materials. J Am Acad Audiol 20: 147–154. 19927677 10.3766/jaaa.20.2.7 PMC2867098

[64] Pichora-Fuller MK, Levitt H (2012) Speech comprehension training and auditory and cognitive processing in older adults. Am J Audiol 21: 351–357. 10.1044/1059-0889(2012/12-0025) 23233521

