# Supplementary figures and images for: Speech Perception in Older Hearing Impaired Listeners: Benefits of Perceptual Training
Source: PLoS One. 2015 Mar 2;10(3):e0113965. doi: 10.1371/journal.pone.0113965 (PMC4346400; doi:10.1371/journal.pone.0113965)

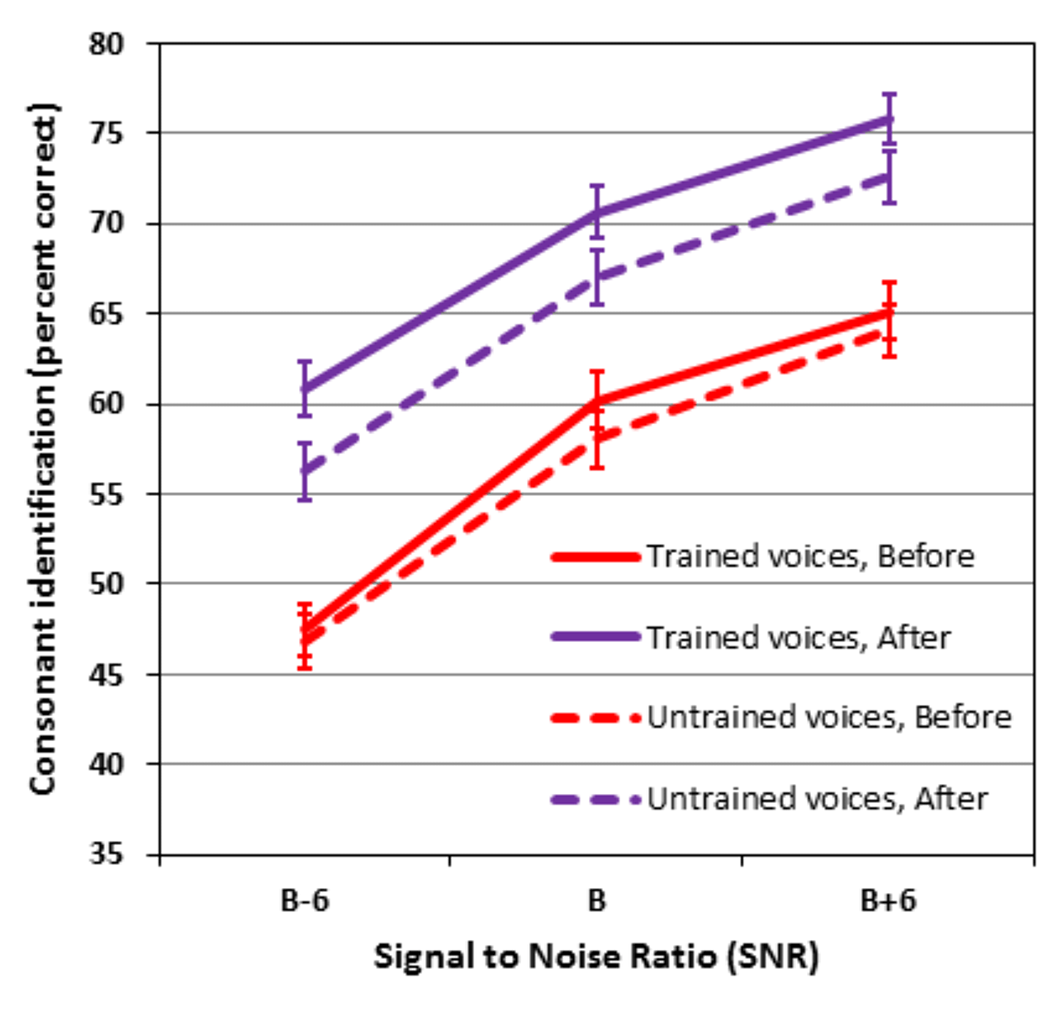

Supplement: S1 Fig — Consonant-identification performance before (red) and after (purple) training for tokens used in training (solid lines) and for tokens spoken by unfamiliar talkers (dashed lines). Percent correct scores are shown at three SNRs, relative to listener-specific and consonant-specific baseline (B). Error bars show standard errors. (TIF) [file pone.0113965.s001.tif]
