# Supplementary material for: Speech Perception in Older Hearing Impaired Listeners: Benefits of Perceptual Training
Source: PLoS One. 2015 Mar 2;10(3):e0113965. doi: 10.1371/journal.pone.0113965 (PMC4346400; doi:10.1371/journal.pone.0113965)
Supplement: S1 Table — Shown are the slopes of the linear regression across training sessions and the correlation of consonant-identification thresholds with training sessions for individual consonants and consonant groups. (DOCX) [file pone.0113965.s002.docx]

| Consonant | Slope (dB/session) | r | F | *p* |
| --- | --- | --- | --- | --- |
| s | -0.43 | 0.95 | 176.65 | 0.0000 |
| z | -0.33 | 0.93 | 119.29 | 0.0000 |
| r | -0.07 | 0.43 | 4.02 | 0.0601 |
| ʃ | -0.43 | 0.93 | 120.91 | 0.0000 |
| ʧ | -0.19 | 0.69 | 15.92 | 0.0009 |
| t | -0.21 | 0.85 | 47.40 | 0.0000 |
| ʤ | -0.43 | 0.87 | 55.74 | 0.0000 |
| l | -0.22 | 0.77 | 25.60 | 0.0001 |
| k | -0.15 | 0.50 | 5.87 | 0.0261 |
| d | -0.13 | 0.50 | 6.10 | 0.0237 |
| g | -0.38 | 0.80 | 31.10 | 0.0000 |
| n | -0.11 | 0.52 | 6.64 | 0.0190 |
| m | -0.10 | 0.61 | 10.45 | 0.0046 |
| f | -0.31 | 0.89 | 67.02 | 0.0000 |
| p | -0.31 | 0.55 | 7.93 | 0.0114 |
| b | -0.34 | 0.59 | 9.51 | 0.0064 |
| h | -0.29 | 0.42 | 3.81 | 0.0667 |
| v | -0.56 | 0.85 | 45.13 | 0.0000 |
| ŋ | -0.68 | 0.80 | 31.71 | 0.0000 |
| θ | -0.66 | 0.80 | 33.00 | 0.0000 |
| ð | -1.15 | 0.96 | 234.63 | 0.0000 |
|  |  |  |  |  |
| Group A | -0.30 | 0.90 | 79.75 | 0.0000 |
| Group B | -0.20 | 0.78 | 27.14 | 0.0001 |
| Group C | -0.58 | 0.84 | 42.85 | 0.0000 |
