# Supplementary material for: Speech Perception in Older Hearing Impaired Listeners: Benefits of Perceptual Training
Source: PLoS One. 2015 Mar 2;10(3):e0113965. doi: 10.1371/journal.pone.0113965 (PMC4346400; doi:10.1371/journal.pone.0113965)
Supplement: S2 Table — Consonant-identification thresholds (dB SNR) before and after training as measured in the laboratory, ANOVA analysis of training effects, and significance of improvement for individual consonants and consonant groups. (DOCX) [file pone.0113965.s003.docx]

| Consonant | Before | After | F[1,15] | *p* |
| --- | --- | --- | --- | --- |
| s | 12.3 | 5.1 | 18.84 | 0.0006 |
| z | 12.3 | 5.8 | 20.44 | 0.0004 |
| r | 5.0 | 1.9 | 11.55 | 0.0040 |
| ʃ | 5.4 | 0.6 | 33.78 | 0.0000 |
| ʧ | 3.8 | 0.7 | 5.63 | 0.0314 |
| t | 14.8 | 8.5 | 17.66 | 0.0008 |
| ʤ | 6.6 | 0.3 | 65.40 | 0.0000 |
| l | 16.7 | 7.9 | 36.58 | 0.0000 |
| k | 16.3 | 9.4 | 13.30 | 0.0024 |
| d | 23.4 | 14.3 | 32.15 | 0.0000 |
| g | 24.6 | 17.0 | 37.27 | 0.0000 |
| n | 23.4 | 17.6 | 8.84 | 0.0095 |
| m | 21.3 | 17.1 | 7.97 | 0.0129 |
| f | 37.8 | 31.9 | 38.70 | 0.0000 |
| p | 30.3 | 18.7 | 13.70 | 0.0021 |
| b | 43.4 | 25.4 | 34.80 | 0.0000 |
| h | 43.1 | 19.5 | 36.50 | 0.0000 |
| v | 54.6 | 41.0 | 22.30 | 0.0003 |
| ŋ | 45.4 | 29.8 | 13.99 | 0.0020 |
| θ | 69.3 | 50.2 | 31.95 | 0.0000 |
| ð | 82.2 | 67.7 | 19.55 | 0.0005 |
|  |  |  |  |  |
| Group A | 8.6 | 3.3 | 46.14 | 0.0000 |
| Group B | 23.5 | 16.4 | 52.09 | 0.0000 |
| Group C | 54.0 | 37.9 | 46.60 | 0.0000 |
