# Supplementary material for: Speech Perception in Older Hearing Impaired Listeners: Benefits of Perceptual Training
Source: PLoS One. 2015 Mar 2;10(3):e0113965. doi: 10.1371/journal.pone.0113965 (PMC4346400; doi:10.1371/journal.pone.0113965)
Supplement: S3 Table — Each row gives the number of consonant responses of each type for the consonant at the top of the column. (DOCX) [file pone.0113965.s004.docx]

|  | b | d | g | r | l | n | m | v | ð | z | ʤ | ʧ | ʃ | s | θ | f | p | t | k | h |
| --- | --- | --- | --- | --- | --- | --- | --- | --- | --- | --- | --- | --- | --- | --- | --- | --- | --- | --- | --- | --- |
| b | 229 | 13 | 8 | 8 | 12 | 2 | 4 | 84 | 3 | 1 | 0 | 0 | 0 | 6 | 16 | 107 | 14 | 3 | 2 | 64 |
| d | 31 | 355 | 31 | 3 | 14 | 0 | 2 | 11 | 10 | 15 | 4 | 2 | 2 | 18 | 20 | 8 | 1 | 10 | 10 | 29 |
| g | 18 | 63 | 340 | 7 | 6 | 1 | 7 | 12 | 6 | 8 | 7 | 3 | 0 | 8 | 5 | 14 | 7 | 5 | 15 | 44 |
| r | 11 | 4 | 4 | 409 | 68 | 3 | 10 | 21 | 5 | 15 | 2 | 0 | 1 | 1 | 0 | 7 | 3 | 2 | 2 | 8 |
| l | 9 | 8 | 3 | 36 | 423 | 6 | 25 | 33 | 3 | 2 | 0 | 0 | 1 | 2 | 2 | 6 | 2 | 2 | 1 | 12 |
| n | 7 | 10 | 0 | 16 | 111 | 321 | 62 | 7 | 6 | 4 | 2 | 0 | 1 | 1 | 2 | 3 | 3 | 2 | 3 | 15 |
| m | 14 | 2 | 1 | 13 | 77 | 46 | 371 | 25 | 1 | 1 | 0 | 0 | 1 | 1 | 2 | 3 | 2 | 0 | 3 | 13 |
| v | 48 | 4 | 6 | 45 | 50 | 5 | 3 | 323 | 33 | 22 | 1 | 0 | 0 | 3 | 5 | 14 | 1 | 2 | 0 | 11 |
| ð | 43 | 42 | 8 | 10 | 106 | 7 | 5 | 144 | 104 | 66 | 0 | 0 | 0 | 2 | 13 | 10 | 0 | 1 | 4 | 11 |
| z | 13 | 45 | 16 | 18 | 34 | 7 | 4 | 24 | 15 | 286 | 45 | 2 | 1 | 25 | 6 | 1 | 5 | 9 | 6 | 14 |
| ʤ | 6 | 39 | 7 | 6 | 7 | 0 | 1 | 3 | 1 | 8 | 373 | 68 | 5 | 5 | 1 | 3 | 1 | 22 | 6 | 14 |
| ʧ | 0 | 2 | 2 | 5 | 2 | 0 | 1 | 0 | 0 | 0 | 29 | 427 | 21 | 6 | 2 | 5 | 2 | 63 | 8 | 1 |
| ʃ | 0 | 7 | 0 | 2 | 1 | 0 | 0 | 0 | 0 | 2 | 22 | 178 | 308 | 17 | 0 | 4 | 2 | 16 | 4 | 13 |
| s | 10 | 22 | 4 | 8 | 7 | 2 | 2 | 11 | 8 | 65 | 7 | 16 | 16 | 278 | 20 | 38 | 2 | 24 | 13 | 23 |
| θ | 20 | 5 | 1 | 3 | 11 | 0 | 1 | 11 | 5 | 15 | 2 | 2 | 1 | 89 | 159 | 187 | 10 | 9 | 6 | 39 |
| f | 32 | 2 | 2 | 2 | 3 | 0 | 4 | 18 | 1 | 0 | 2 | 4 | 5 | 28 | 54 | 324 | 25 | 11 | 5 | 54 |
| p | 4 | 4 | 2 | 2 | 3 | 1 | 1 | 3 | 2 | 0 | 0 | 4 | 0 | 2 | 5 | 21 | 337 | 44 | 38 | 103 |
| t | 6 | 6 | 3 | 1 | 6 | 0 | 3 | 3 | 4 | 4 | 10 | 26 | 3 | 9 | 13 | 10 | 35 | 337 | 38 | 59 |
| k | 1 | 1 | 8 | 3 | 0 | 0 | 1 | 2 | 1 | 0 | 2 | 13 | 0 | 5 | 5 | 11 | 34 | 41 | 400 | 48 |
| h | 6 | 0 | 5 | 1 | 4 | 4 | 2 | 1 | 1 | 0 | 1 | 8 | 0 | 4 | 8 | 41 | 39 | 13 | 40 | 398 |
