# Supplementary material for: Speech Perception in Older Hearing Impaired Listeners: Benefits of Perceptual Training
Source: PLoS One. 2015 Mar 2;10(3):e0113965. doi: 10.1371/journal.pone.0113965 (PMC4346400; doi:10.1371/journal.pone.0113965)
Supplement: S4 Table — Each row gives the number of consonant responses of each type for the consonant at the top of the column. (DOCX) [file pone.0113965.s005.docx]

|  | b | d | g | r | l | n | m | v | ð | z | ʤ | ʧ | ʃ | s | θ | f | p | t | k | h |
| --- | --- | --- | --- | --- | --- | --- | --- | --- | --- | --- | --- | --- | --- | --- | --- | --- | --- | --- | --- | --- |
| b | 348 | 3 | 3 | 6 | 8 | 0 | 6 | 72 | 6 | 1 | 0 | 0 | 0 | 1 | 21 | 66 | 1 | 0 | 1 | 33 |
| d | 25 | 394 | 42 | 4 | 4 | 1 | 1 | 11 | 14 | 4 | 4 | 2 | 1 | 5 | 18 | 11 | 2 | 5 | 7 | 21 |
| g | 19 | 41 | 406 | 5 | 8 | 6 | 5 | 10 | 1 | 1 | 3 | 1 | 0 | 0 | 10 | 6 | 5 | 1 | 10 | 38 |
| r | 4 | 4 | 7 | 436 | 51 | 7 | 11 | 24 | 5 | 5 | 2 | 0 | 1 | 2 | 1 | 8 | 1 | 1 | 3 | 3 |
| l | 4 | 0 | 0 | 37 | 465 | 17 | 20 | 17 | 8 | 1 | 0 | 0 | 0 | 0 | 3 | 1 | 0 | 0 | 0 | 3 |
| n | 1 | 3 | 4 | 9 | 69 | 389 | 80 | 5 | 2 | 2 | 1 | 1 | 0 | 0 | 0 | 0 | 1 | 0 | 0 | 9 |
| m | 5 | 1 | 7 | 16 | 58 | 70 | 406 | 4 | 1 | 1 | 0 | 0 | 0 | 1 | 0 | 0 | 1 | 0 | 1 | 4 |
| v | 22 | 2 | 10 | 14 | 30 | 4 | 8 | 420 | 28 | 9 | 0 | 0 | 0 | 0 | 6 | 16 | 1 | 0 | 0 | 6 |
| ð | 25 | 20 | 5 | 17 | 76 | 3 | 5 | 188 | 172 | 23 | 0 | 1 | 0 | 1 | 19 | 13 | 0 | 0 | 0 | 8 |
| z | 4 | 24 | 17 | 13 | 18 | 8 | 1 | 34 | 30 | 351 | 30 | 5 | 1 | 17 | 4 | 2 | 0 | 5 | 3 | 9 |
| ʤ | 1 | 19 | 8 | 1 | 2 | 0 | 0 | 1 | 2 | 9 | 426 | 65 | 5 | 1 | 0 | 1 | 2 | 18 | 9 | 6 |
| ʧ | 0 | 0 | 0 | 1 | 0 | 0 | 0 | 1 | 0 | 0 | 14 | 478 | 44 | 3 | 1 | 0 | 1 | 29 | 2 | 2 |
| ʃ | 1 | 0 | 0 | 0 | 0 | 0 | 0 | 0 | 0 | 3 | 10 | 110 | 417 | 13 | 3 | 1 | 1 | 4 | 4 | 9 |
| s | 9 | 10 | 15 | 4 | 2 | 3 | 2 | 3 | 6 | 59 | 18 | 22 | 16 | 307 | 30 | 21 | 3 | 14 | 15 | 17 |
| θ | 29 | 5 | 2 | 0 | 2 | 0 | 0 | 9 | 9 | 2 | 0 | 1 | 1 | 33 | 262 | 190 | 6 | 1 | 2 | 22 |
| f | 48 | 0 | 1 | 2 | 1 | 2 | 1 | 22 | 3 | 1 | 0 | 2 | 3 | 18 | 49 | 345 | 15 | 4 | 6 | 53 |
| p | 6 | 2 | 2 | 0 | 0 | 0 | 2 | 0 | 1 | 0 | 0 | 2 | 0 | 0 | 2 | 11 | 365 | 15 | 28 | 140 |
| t | 3 | 7 | 2 | 1 | 1 | 1 | 2 | 1 | 1 | 6 | 10 | 27 | 2 | 3 | 7 | 6 | 43 | 346 | 41 | 66 |
| k | 1 | 3 | 5 | 3 | 2 | 0 | 2 | 0 | 0 | 0 | 0 | 2 | 0 | 0 | 1 | 10 | 20 | 25 | 416 | 86 |
| h | 4 | 1 | 1 | 0 | 4 | 0 | 0 | 4 | 0 | 0 | 1 | 2 | 0 | 1 | 2 | 14 | 28 | 3 | 15 | 496 |
