# Supplementary material for: Speech Perception in Older Hearing Impaired Listeners: Benefits of Perceptual Training
Source: PLoS One. 2015 Mar 2;10(3):e0113965. doi: 10.1371/journal.pone.0113965 (PMC4346400; doi:10.1371/journal.pone.0113965)
Supplement: S5 Table — Each row gives the number of consonant responses of each type for the consonant at the top of the column. (DOCX) [file pone.0113965.s006.docx]

|  | b | d | g | r | l | ŋ | n | m | v | ð | z | ʤ | ʧ | ʃ | s | θ | f | p | t | k |
| --- | --- | --- | --- | --- | --- | --- | --- | --- | --- | --- | --- | --- | --- | --- | --- | --- | --- | --- | --- | --- |
| b | 376 | 59 | 17 | 0 | 0 | 1 | 4 | 14 | 83 | 5 | 2 | 2 | 0 | 0 | 0 | 0 | 1 | 10 | 1 | 1 |
| d | 41 | 408 | 32 | 1 | 2 | 1 | 5 | 5 | 32 | 13 | 1 | 11 | 0 | 0 | 1 | 6 | 5 | 7 | 4 | 1 |
| g | 67 | 108 | 287 | 0 | 1 | 1 | 9 | 10 | 51 | 14 | 3 | 2 | 1 | 0 | 1 | 2 | 5 | 7 | 4 | 3 |
| r | 13 | 19 | 20 | 354 | 28 | 5 | 18 | 10 | 34 | 3 | 9 | 14 | 7 | 8 | 6 | 1 | 10 | 6 | 7 | 4 |
| l | 10 | 11 | 14 | 40 | 342 | 11 | 23 | 26 | 72 | 5 | 5 | 3 | 0 | 0 | 3 | 0 | 4 | 3 | 1 | 3 |
| ŋ | 4 | 11 | 12 | 5 | 12 | 205 | 146 | 130 | 36 | 3 | 5 | 1 | 0 | 0 | 0 | 0 | 0 | 0 | 4 | 2 |
| n | 7 | 15 | 5 | 1 | 7 | 24 | 376 | 112 | 23 | 3 | 2 | 0 | 0 | 1 | 0 | 0 | 0 | 0 | 0 | 0 |
| m | 12 | 3 | 3 | 3 | 7 | 21 | 95 | 405 | 19 | 0 | 5 | 0 | 0 | 1 | 0 | 0 | 1 | 1 | 0 | 0 |
| v | 41 | 15 | 12 | 7 | 11 | 6 | 19 | 28 | 400 | 14 | 11 | 1 | 0 | 0 | 2 | 2 | 5 | 1 | 0 | 1 |
| ð | 29 | 83 | 18 | 6 | 7 | 2 | 18 | 25 | 270 | 84 | 22 | 6 | 0 | 0 | 1 | 1 | 1 | 2 | 0 | 1 |
| z | 19 | 35 | 25 | 3 | 8 | 5 | 20 | 9 | 74 | 22 | 273 | 49 | 11 | 2 | 10 | 0 | 7 | 0 | 2 | 2 |
| ʤ | 15 | 47 | 23 | 5 | 1 | 3 | 4 | 1 | 7 | 3 | 6 | 423 | 22 | 1 | 1 | 4 | 1 | 1 | 3 | 5 |
| ʧ | 2 | 4 | 2 | 1 | 0 | 1 | 3 | 5 | 4 | 0 | 2 | 32 | 437 | 16 | 8 | 2 | 7 | 5 | 18 | 27 |
| ʃ | 0 | 2 | 5 | 0 | 2 | 2 | 2 | 5 | 2 | 3 | 4 | 30 | 56 | 399 | 25 | 12 | 4 | 1 | 9 | 13 |
| s | 8 | 9 | 14 | 1 | 4 | 2 | 8 | 4 | 13 | 1 | 18 | 13 | 31 | 56 | 259 | 33 | 39 | 19 | 28 | 16 |
| θ | 10 | 12 | 4 | 4 | 0 | 1 | 3 | 3 | 17 | 8 | 3 | 1 | 2 | 8 | 50 | 192 | 172 | 13 | 45 | 28 |
| f | 8 | 4 | 6 | 1 | 3 | 1 | 1 | 0 | 22 | 2 | 2 | 2 | 2 | 11 | 32 | 81 | 322 | 23 | 34 | 19 |
| p | 34 | 7 | 1 | 0 | 0 | 0 | 1 | 1 | 2 | 0 | 0 | 0 | 3 | 1 | 1 | 14 | 28 | 313 | 75 | 95 |
| t | 1 | 12 | 6 | 0 | 2 | 0 | 1 | 2 | 4 | 1 | 3 | 3 | 23 | 4 | 8 | 19 | 15 | 36 | 379 | 57 |
| k | 2 | 5 | 14 | 1 | 2 | 1 | 0 | 2 | 3 | 0 | 0 | 1 | 6 | 2 | 2 | 13 | 21 | 38 | 76 | 387 |
