# Supplementary material for: Speech Perception in Older Hearing Impaired Listeners: Benefits of Perceptual Training
Source: PLoS One. 2015 Mar 2;10(3):e0113965. doi: 10.1371/journal.pone.0113965 (PMC4346400; doi:10.1371/journal.pone.0113965)
Supplement: S6 Table — Each row gives the number of consonant responses of each type for the consonant at the top of the column. (DOCX) [file pone.0113965.s007.docx]

|  | b | d | g | r | l | ŋ | n | m | v | ð | z | ʤ | ʧ | ʃ | s | θ | f | p | t | k |
| --- | --- | --- | --- | --- | --- | --- | --- | --- | --- | --- | --- | --- | --- | --- | --- | --- | --- | --- | --- | --- |
| b | 385 | 60 | 24 | 0 | 1 | 1 | 2 | 7 | 71 | 11 | 1 | 1 | 0 | 0 | 0 | 0 | 3 | 9 | 0 | 0 |
| d | 27 | 432 | 21 | 1 | 1 | 1 | 6 | 3 | 23 | 36 | 3 | 7 | 0 | 0 | 2 | 1 | 1 | 4 | 5 | 2 |
| g | 52 | 73 | 352 | 1 | 0 | 0 | 6 | 7 | 48 | 19 | 5 | 3 | 0 | 0 | 0 | 3 | 0 | 1 | 1 | 5 |
| r | 1 | 4 | 3 | 396 | 73 | 1 | 9 | 4 | 16 | 3 | 14 | 15 | 15 | 9 | 4 | 3 | 1 | 1 | 1 | 3 |
| l | 2 | 3 | 6 | 38 | 462 | 8 | 6 | 14 | 23 | 3 | 5 | 2 | 0 | 0 | 1 | 1 | 1 | 0 | 0 | 1 |
| ŋ | 1 | 4 | 6 | 0 | 16 | 314 | 127 | 82 | 18 | 4 | 3 | 1 | 0 | 0 | 0 | 0 | 0 | 0 | 0 | 0 |
| n | 3 | 3 | 2 | 1 | 7 | 47 | 422 | 71 | 13 | 3 | 3 | 0 | 0 | 1 | 0 | 0 | 0 | 0 | 0 | 0 |
| m | 1 | 1 | 2 | 0 | 5 | 45 | 92 | 413 | 12 | 1 | 2 | 0 | 0 | 0 | 0 | 1 | 0 | 0 | 0 | 1 |
| v | 32 | 9 | 12 | 1 | 13 | 3 | 16 | 21 | 411 | 45 | 7 | 3 | 0 | 0 | 0 | 0 | 1 | 1 | 1 | 0 |
| ð | 29 | 50 | 14 | 1 | 9 | 4 | 14 | 10 | 203 | 219 | 17 | 3 | 1 | 0 | 0 | 2 | 0 | 0 | 0 | 0 |
| z | 5 | 10 | 19 | 8 | 9 | 2 | 7 | 3 | 44 | 37 | 360 | 42 | 8 | 3 | 9 | 2 | 2 | 1 | 4 | 1 |
| ʤ | 1 | 10 | 4 | 0 | 2 | 0 | 0 | 0 | 3 | 4 | 3 | 532 | 11 | 1 | 0 | 1 | 0 | 0 | 3 | 1 |
| ʧ | 1 | 1 | 0 | 0 | 0 | 0 | 0 | 0 | 0 | 2 | 1 | 58 | 463 | 32 | 3 | 0 | 1 | 1 | 12 | 1 |
| ʃ | 0 | 2 | 1 | 2 | 1 | 0 | 0 | 0 | 1 | 0 | 3 | 34 | 52 | 447 | 15 | 9 | 6 | 0 | 1 | 2 |
| s | 3 | 13 | 8 | 10 | 3 | 0 | 2 | 0 | 7 | 3 | 13 | 15 | 33 | 37 | 337 | 40 | 18 | 5 | 17 | 12 |
| θ | 3 | 4 | 5 | 2 | 4 | 0 | 0 | 0 | 13 | 4 | 1 | 2 | 0 | 1 | 28 | 311 | 155 | 15 | 21 | 7 |
| f | 5 | 1 | 0 | 0 | 5 | 0 | 1 | 3 | 22 | 1 | 2 | 2 | 0 | 5 | 17 | 114 | 348 | 24 | 15 | 11 |
| p | 26 | 2 | 5 | 0 | 0 | 0 | 0 | 0 | 5 | 0 | 0 | 0 | 0 | 0 | 0 | 11 | 19 | 392 | 63 | 53 |
| t | 2 | 7 | 9 | 1 | 1 | 0 | 1 | 1 | 1 | 0 | 0 | 5 | 16 | 3 | 4 | 16 | 6 | 37 | 441 | 25 |
| k | 3 | 2 | 8 | 0 | 0 | 0 | 1 | 1 | 1 | 0 | 0 | 4 | 2 | 0 | 0 | 23 | 18 | 61 | 55 | 397 |
